# On the Selective Enzymatic Recycling of Poly(pentamethylene 2,5-furanoate)/Poly(lactic acid) Blends and Multiblock Copolymers

**DOI:** 10.1021/acssuschemeng.3c01796

**Published:** 2023-06-16

**Authors:** Chiara Siracusa, Felice Quartinello, Michelina Soccio, Mattia Manfroni, Nadia Lotti, Andrea Dorigato, Georg M. Guebitz, Alessandro Pellis

**Affiliations:** † acib GmbH, Konrad-Lorenz-Strasse 20, 3430 Tulln, Donau, Austria; ‡ Institute of Environmental Biotechnology, University of Natural Resources and Life Sciences Vienna Konrad-Lorenz-Strasse 20, 3430 Tulln, Donau, Austria; § Department of Civil, Chemical, Environmental and Materials Engineering (DICAM), University of Bologna, Bologna 40138, Italy; ∥ Interdepartmental Center for Industrial Research on Advanced Applications in Mechanical Engineering and Materials Technology, CIRI-MAM, University of Bologna, Bologna 40138, Italy; ⊥ Interdepartmental Center for Agro-Food Research, CIRI-AGRO, University of Bologna, Bologna 40126, Italy; # Department of Industrial Engineering and INSTM Research Unit, University of Trento, Trento 38123, Italy; ∇ Department of Chemistry and Industrial Chemistry, Università degli Studi di Genova, Via Dodecaneso 31, 16146 Genova, Italy

**Keywords:** enzymatic depolymerization, 2,5-furandicarboxylic acid, poly(lactic acid), poly(pentamethylene 2,5-furandicarboxylate), monomers recovery, polymer resynthesis

## Abstract

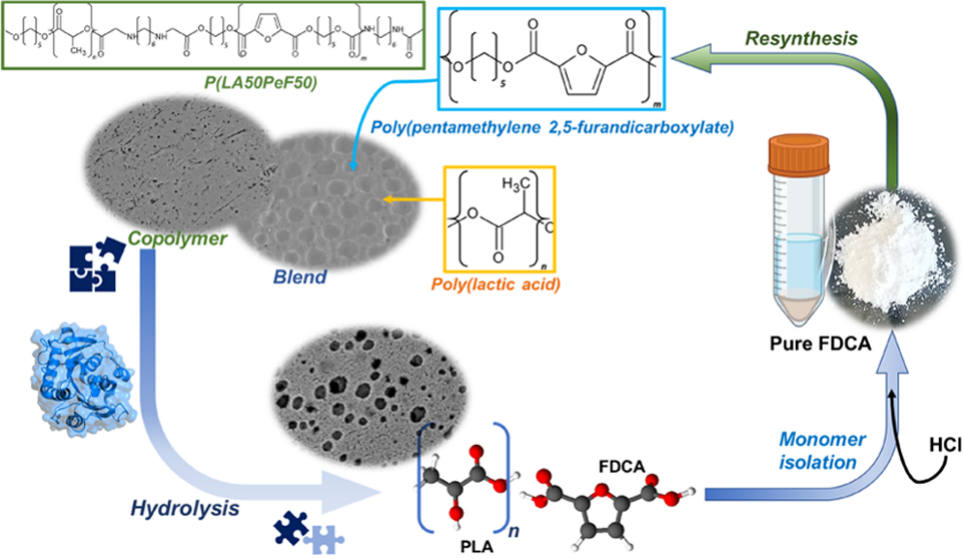

Among novel renewable furanoate-based polyesters, poly(pentamethylene 2,5-furandicarboxylate) (PPeF) shows outstanding gas barrier properties and high flexibility. PPeF blending/copolymerization with another well-known bio-based polymer, poly(lactic acid) (PLA), leads to considerably better mechanical and gas barrier properties of the latter, making it suitable for flexible food packaging applications. In this work, enzymatic depolymerization of PLA/PPeF blends with different compositions (1, 3, 5, 20, 30, and 50 wt % PPeF) and a PLA-PPeF block copolymer (50 wt % PPeF) by cutinase 1 from *Thermobifida cellulositilytica* (Thc_Cut1) was investigated as a possible recycling strategy. Based on quantification of weight loss and high-performance liquid chromatography (HPLC) analysis of released molecules, faster hydrolysis was seen for PLA/PPeF blends with increasing PPeF content when compared to neat PLA, while the block copolymer (P(LA50PeF50)) was significantly less susceptible to hydrolysis. Surface morphology analysis (via scanning electron microscopy), Fourier transform infrared spectroscopy, and NMR analysis confirmed preferential hydrolysis of the PPeF component. Through crystallization, 2,5-furandicarboxylic acid was selectively recovered from the depolymerized films and used for the resynthesis of the PPeF homopolymer, demonstrating the potential of enzymes for novel recycling concepts. The possibility of selective recovery of 2,5-furandicarboxylic acid from the completely depolymerized films with a 75% yield could bring further evidence of the high value of these materials, both in the form of blends and copolymers, for a sustainable whole packaging life cycle, where PPeF is potentially enzymatically recycled and PLA is mechanically recycled.

## Abstract 2

This work developed a sustainable plastic recycling approach that allows the selective depolymerization of one component of a blend and the monomer recovery for the subsequent resynthesis process.

## Introduction

The industrial sector in which plastics are most widely used in Europe is rigid and flexible packaging, accounting for around 40% of the total market share. Despite the lightness and all of the other well-known advantages of synthetic polymers, the total environmental impact is astonishing. Each European inhabitant is in fact estimated to generate 34.6 kg of packaging waste per year.^1^

Recently, the European Union has been tackling this issue by introducing restrictions to certain types of single-use items such as plastic cutlery and plates, as well as straws.^2^ Moreover, innovative strategies for recycling are also being investigated and implemented to close the plastic carbon cycle.^3^ Nevertheless, there is an increasing need for novel bio-based materials with comparable performance to traditional plastics, but at the same time with intrinsic recycling properties and cost-effectiveness.

These raised awareness of the plastic problem with consequent growing demand for bio-based and biodegradable plastics, leading to new formulations mainly represented by rigid and flexible packaging.^4,5^ Polymers based on 2,5-furandicarboxylic acid (FDCA), such as poly(ethylene 2,5-furandicarboxylate) (PEF),^6^ poly(propylene 2,5-furanoate) (PPF)^7,8^ poly(butylene 2,5-furandicarboxylate) (PBF);^9−11^ poly(pentamethylene 2,5-furandicarboxylate) (PPeF),^12−15^ and poly(hexamethylene 2,5-furandicarboxylate) (PHF),^15,16^ have great potential as alternatives to poly(ethylene terephthalate) (PET) containing fossil-based terephthalic acid (TA). FDCA can be produced from renewable resources^17^ involving chemical and/or enzymatic strategies.^18^ Synthesis of PEF is currently upscaled to an industrial level for the production of bottles.^19^ The glass-transition temperature, as well as the mechanical and permeability properties of PEF, are comparable to or even better than those of PET.^20^ The furan ring, presenting lower aromaticity and a less linear and less flexible structure when compared to terephthalic acid, leads to and provides an overall lower covalent strength of polymers.^5^ Although the susceptibility of PEF to enzymatic hydrolysis was demonstrated,^19^ overall biodegradability seems to be low.^21,22^ Therefore, the potential of blending, copolymerization, or additive addition to improve the performance of FDCA-based materials is investigated.^23^

Poly(lactic acid) (PLA) is the only 100% bio-based alternative to conventional polyesters currently available on the market in larger quantities.^23,24^ PLA is biocompatible and available at an affordable cost but finds some major limitations issues due to its brittleness and low thermal stability.^25,26^

Moreover, while PLA has been defined as fully biodegradable, fast degradation only occurs under industrial composting conditions. Several copolymers and blends were therefore developed to improve its properties.^18^ Blends with PET have been investigated to improve thermomechanical properties,^27^ which, however, raise concerns due to the fossil origin and recalcitrance of PET.

On the other hand, PPeF specifically represents an interesting candidate for PLA-based blends and copolymers, and promising properties for packaging applications have been demonstrated. The combination with PPeF overcomes intrinsic PLA brittleness and allows the improvement of its gas barrier properties,^25,28^ meanwhile not affecting the transparency of the cast films.^17^ More in detail, both physical and chemical blending of PLA and PPeF leads to an improvement of the mechanical response in terms of flexibility without compromising the O_2_ and CO_2_ barrier properties that keep better than polyolefins,^25,28^ making PLA/PPeF materials suitable for flexible food packaging applications. In this work, for the first time, the potential of enzymatic depolymerization of various PLA/PPeF materials (physical blends and block copolymer) has been investigated, which could open new recycling possibilities for the recovery of valuable building blocks.

The susceptibility of PLA to hydrolysis by enzymes seems to be limited, while copolymer formulations increased hydrolysis rates.^29^ This suggests that blending PLA with PPeF could also improve the overall biodegradability of the resulting material.

## Materials and Methods

**Scheme 1:**
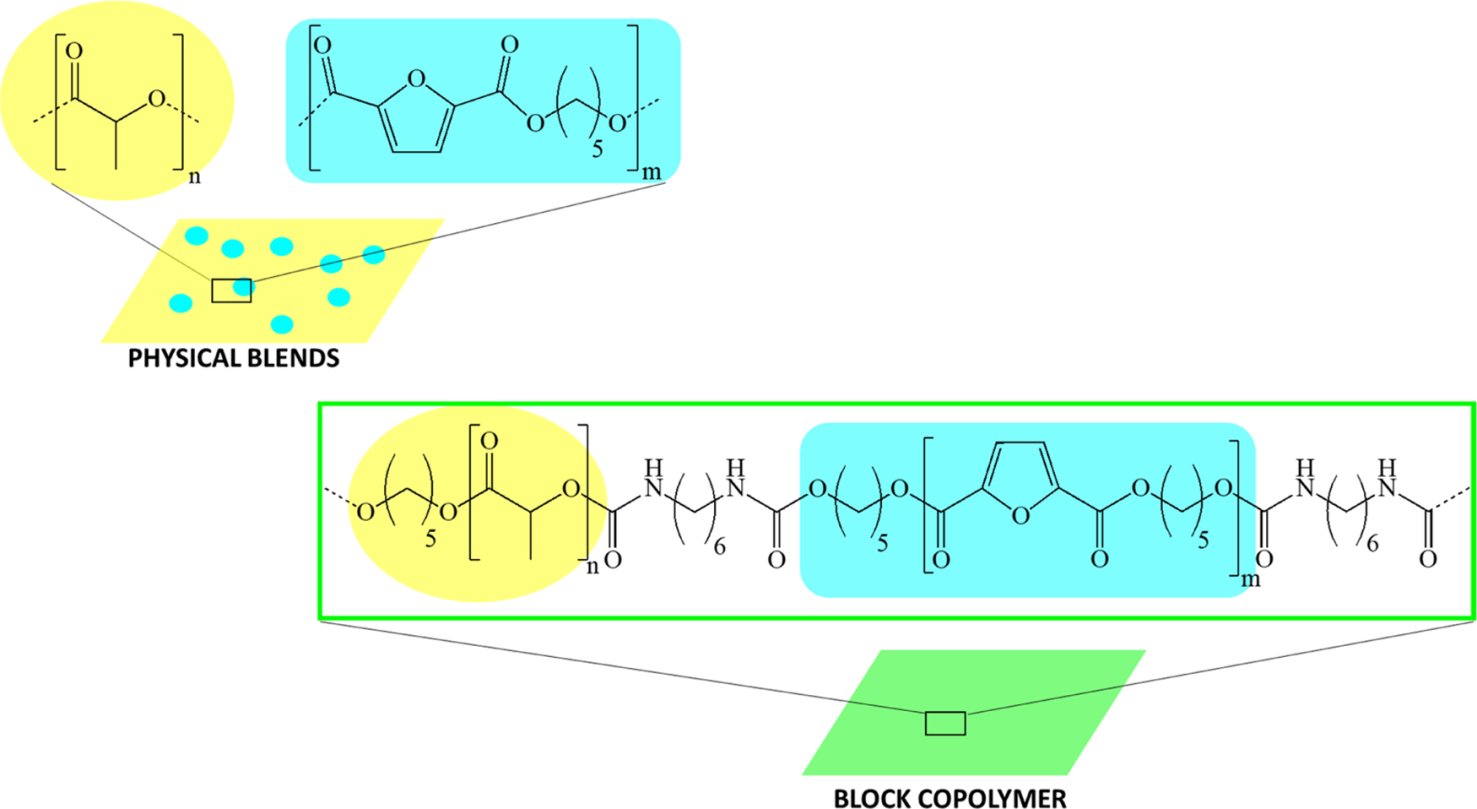
Representation of the PLA/PPeF Physical Blend (Top) and Block Copolymer (Bottom) Chemical Structure and Architecture

Various synthetic polyesters based on the chemical^28^ or physical^25^ combination of poly(lactic acid) (PLA) and poly(1,5-pentamethylene 2,5-furanoate) (PPeF) were prepared and processed in the form of thin films (≈150 μm). The covalent formulation was a 50 wt % block copolymer of PLA (kindly provided by CORBION) and PPeF connected with a diamine chain extender (see the SI, Scheme S1) that from now on will be labeled as P(LA50PeF50). The physical blends ranged from 1 to 50 wt % (namely, 1, 3, 5, 20, 30, and 50 wt %) of the PPeF mass percentage, from now on labeled as PP1, PP3, etc. based on PPeF wt %. Scheme 1 shows the chemical structure and architecture of the physical blends (top) and the block copolymer (bottom).

Dipotassium hydrogen phosphate (K_2_HPO_4_) was obtained from Roth (Germany). All other chemicals were purchased from Sigma-Aldrich. The used enzyme was thermostable cutinase 1 from *Thermobifida cellulosityca* (Thc_Cut1) expressed, purified, and then characterized in terms of concentration and activity as previously reported.^30^

### Enzyme Characterization: Protein Concentration and Activity Assay

Enzyme concentration in the stock solution was determined via the Bradford assay. 10 μL of the diluted sample and water/buffer as blanks were placed in triplicate in a transparent 96-well Greiner plate. The addition of 200 μL of the diluted Bradford reagent was followed by a 5-min room-temperature incubation while shaking. A Tecan plate reader was then used to measure the absorbance at 595 nm and the concentration was subsequently calculated referring to bovine serum albumin protein as the standard.

*para*-Nitrophenylbutyrate (*p*-NPB) was used as a substrate for the cutinase activity assay. 20 μL of the enzyme (prediluted in ultrapure water to 1:100 and 1:1000) were placed in triplicate in a transparent 96-well plate. Then, 200 μL of the substrate (diluted in butanol and buffer of incubation) was added to both the enzyme dilutions and the blanks (water/buffer). The absorbance at 405 nm was measured for 10 min at 25 °C every 18 seconds. The activity was expressed in units, corresponding to the amount of enzyme necessary to hydrolyze 1 μmol of substrate per minute.

### Film Preparation and Enzymatic Treatment

The polymers were cut in 0.5 cm × 1.0 cm strips (average weight 3.09 ± 0.61 mg) and washed in three subsequent steps to remove surface impurities: Triton X-100, Na_2_CO_3_, and a final rinsing with ultrapure water.

The enzymatic solution was prepared in 1 M KPO buffer pH 8 (K_2_HPO_4_/KH_2_PO_4_) to a final concentration of 5 μM. The films were horizontally incubated in Eppendorf tubes with 2 mL of enzyme dilution at 65 °C under agitation (150 rpm). The reaction was monitored throughout the incubation course, collecting samples after 3, 6, and 7 days to follow the degradation pattern. The reaction was stopped at the different time points by cooling down to 4 °C, a temperature at which the enzyme activity is negligible. The enzyme was then removed from the hydrolysate through ice-cold methanol addition. After acidification (20 μL 6 N HCl) the samples were centrifuged (14,000 rpm, 4 °C, 15 min) and filtered (0.2 μM polyamide filters) to further clean the solution from the residual enzymatic content. At the same time, a set of 3 blanks per each film type was incubated with buffer only as controls. For each time point, experiments were performed in triplicate.

### Weight Loss and Surface Characterization of the Polymers (FT-IR and SEM)

For those samples with a weighable residual portion, the weight loss was measured and compared to the initial one after washing and drying the recovered film. This served as a first evaluation of the efficacy of enzymatic treatment, Fourier transform infrared spectroscopy (FT-IR) was used to characterize the surface functional groups using a PerkinElmer Spectrum 100 FT-IR spectrometer in ATR mode. Spectra were recorded from 650 to 4000 cm^–1^ for 40 scans (resolution 2 cm^–1^). Scanning electron microscopy (SEM) images were acquired using a Hitachi 3030 at a set of increasing magnifications (100×, 500×, 1000×, and 2500×). Images were acquired after applying a 3 nm platinum coating.

### Quantification of Hydrolysis Products

The recovered hydrolysates after each time point were quantified for the released monomers via high-performance liquid chromatography (HPLC) instruments (Agilent Technologies, 1260 Infinity) equipped with reversed-phase column C18. For the aromatic compound FDCA, a UV detector was used; the gradient was based on methanol and 0.1% formic acid at a flow rate of 0.85 mL min^–1^, while the injected volume was 10 μL. The equilibration of the column to the initial gradient was carried out after each run for a total of 20-minute run per sample. The preparation of the samples for the HPLC analysis included a methanol-based precipitation protocol aiming at the removal of the enzyme from the solution. In short, samples were diluted in ice-cold methanol and 20 μL of 6 N HCl was added. Centrifugation for 15 min (14000 rpm, 4 °C) was performed and the isolated supernatant was subsequently filtered (0.2 μm polyamide filters) into HPLC vials.

For the quantification of aliphatic molecules like lactic acid, a refractive index detector was used. HPLC analysis samples were cleaned from proteins through Carrez clarification based on potassium hexacyanoferrate(II) trihydrate and zinc sulfate heptahydrate reagents. The two reagents were added sequentially to the hydrolysates and allowed to rest, respectively, for 1 and 10 min. A centrifugation step was also performed (30 min, 1400 rpm, 4 °C) before filtering the clear supernatant into an HPLC vial. HPLC in use was coupled with a refractive index detector (Transgenomic IC SEP-ION-300) and run in 0.01 N H_2_SO_4_, with a flow rate of 0.325 mL min^–1^, 45 °C. Concentrations of hydrolysis products were calculated based on calibration curves of both FDCA and lactic acid standards, prepared and measured as the samples (see the SI, Figure S1A,B).

### Monomer Recovery and Purification

An amount of 1.5 g of the PPeF homopolymer was incubated for 72 h in 50 mL of 1 M KPO buffer and 5 μM Thc_cut1. The hydrolysate was acidified with 6 N HCl. The amount of acid added was adjusted according to the sample’s initial acidity to reach approximately a pH of 2. After vortexing the solution, a centrifugation step (30 min, 3200 rpm) was performed until a clear separation between the precipitate and the supernatant could be seen. The precipitate was resuspended in ultrapure water, acidified with 6 N HCl (to pH = 2), centrifuged, and isolated from the supernatant with the procedure already described. The separated pellet was further washed with ultrapure water and then freeze-dried and analyzed via HPLC and FT-IR. Thermogravimetric analysis (TGA) was carried out using a 20 mL min^–1^ N_2_ flow with 10 mg of each sample that was heated from 25 to 900 °C using a heat rate of 10 °C per min using a Netzsch TG instrument 209 F1. The supernatant was kept for further characterization and isolation of the residual monomer.

### ^1^H NMR Analysis of Hydrolysates and Recovered Materials

The differential hydrolysis of the polymers was monitored through ^1^H NMR analysis of the main time-point hydrolyzed samples and compared to the spectra of each blank. ^1^H NMR spectra were recorded on a JEOL ECZ400R/S3 spectrometer (400 MHz for ^1^H). CDCl_3_ was used as the NMR solvent if not otherwise specified.

The same analytic technique was applied to the recovered monomer (FDCA) to gain further information about purity. DMSO was used as a solvent, and the spectra of different stages of purification were compared with those of pure commercial FDCA (Sigma-Aldrich).

### Resynthesis of PPeF (R-PPeF)

Resynthesized poly(1,5-pentamethylene 2,5-furanoate) (R-PPeF) was prepared by two-stage polycondensation synthesis. The reaction was conducted in a 200 mL thermostatted stirred reactor, in which the reagents, R-FDCA and 1,5-pentanediol (PD), were placed together with the catalysts, titanium tetrabutoxide (TBT) and titanium isopropoxide (TTIP) (Table S3). To promote the solubilization of the diacid and consequently the esterification reaction, a 300% molar excess of glycol was employed (Table S3). The first stage was carried out for 1.5 h at 190–195 °C under reflux and a nitrogen atmosphere by stirring at 50 rpm; afterward, the condenser was removed, and the gas flow was increased to allow the distillation of water for 1 additional hour. Afterward, the reaction vessel was gradually heated to 220 °C and the pressure was simultaneously reduced to 0.01 mbar. The second stage lasted until a constant value of torque was reached, which required 2 h. Starting from 0.484 g (0.0031 mol) of R-FDCA, 0.620 g (0.0028) of R-PPeF was obtained (yield 90%).

### Molecular Characterization

^1^H NMR analysis was performed to check the chemical structure of the resynthesized polymer. The ^1^H NMR spectrum was recorded on a Varian XL-400 NMR spectrometer (Palo Alto, CA) at room temperature (relaxation time = 0 s, acquisition time = 1 s, 100 repetitions). The polymeric solution was prepared by dissolving 10 mg of R-PPeF in deuterated chloroform (CDCl_3_, containing 0.03% TMS as an internal reference) with a concentration of 0.5 wt %.

Molecular characterization was also implemented by attenuated total reflectance–Fourier transform infrared spectroscopy (PerkinElmer ATR-FT-IR spectrometer Spectrum One). The measurements were performed in a 450–4000 cm^–1^ wavelength range at a 4 cm^–1^ resolution, with 32 scans, and processed by a PerkinElmer data manager (Spectrum).

To determine polymer molecular weight, GPC analysis was carried out at 30 °C with an HPLC Lab Flow 2000 apparatus (KNAUER, Berlin, Germany) equipped with a Rheodyne 7725i injector, a Phenomenex MXL 5 μm mixed bed column, and a RI K-2301 KNAUER detector. The mobile phase used was chloroform with the flow rate fixed at 1.0 mL min^–1^ and the injected solutions had a concentration of 2 mg mL^–1^. Monodisperse polystyrene standards (Sigma-Aldrich Chemical Co., St. Louis, MO) were used for the calibration curve.

### Thermal Characterization

DSC analysis was performed to determine the main thermal transition in the polymer when subjected to a predetermined heating program. Measurements were conducted with a Pyris DSC6 calorimeter (PerkinElmer, Shelton, CT) under nitrogen flux (20 mL min^–1^) using the following thermal program. The polymer was brought to −70 °C and heated up to 200 °C at 20 °C min^–1^ (I scan). The glass-transition temperature (*T*_g_) was calculated as the midpoint of the glass-to-rubber transition step, while the specific heat increment (Δ*C*_p_) was obtained from the jump height between the two baselines associated with the glass-transition step.

Thermogravimetric analysis (PerkinElmer TGA7), was employed to evaluate the polymer’s thermal stability by heating 5 mg of the polymer from 40 to 800 °C at 10 °C min^–1^ under a nitrogen atmosphere (gas flow: 40 mL min^–1^). The temperature of initial degradation (*T*_onset_) and the temperature corresponding to the maximum degradation rate (*T*_max_) were determined.

### Film Preparation

Polymer films (150 μm thickness) were obtained by compression molding into Teflon sheets at 100 °C with a laboratory press Carver C12 under a pressure of 3.0 ton m^–2^. Then, the films were stored at room temperature for 10 days before further characterization.

### Mechanical Characterization

R-PPeF rectangular specimens (50 mm × 5 mm) were characterized from a mechanical point of view through quasi-static tensile tests to evaluate the elastic modulus (*E*), elongation at break (ε_b_), and stress at break (σ_b_). After having measured the average thickness, the samples were fixed to the instrument, an Instron 5966 dynamometer (Norwood, MA) equipped with a 10 kN load cell. A 20 mm gauge length was used. The tests were conducted at room temperature with a testing speed of 10 mm min^–1^.

## Results and Discussion

### Enzymatic Hydrolysis of the PPeF/PLA Blends and Copolymer

The enzyme recombinantly produced for PLA/PPeF blend hydrolysis, namely, Thc_Cut1, had a total protein concentration of 5 mg mL^–1^ and an activity of 184 U mg^–1^ on *p*-NPB. The films were all incubated with the recombinant Thc_cut1 for 3, 6, and 7 days to investigate the degradation pattern. Optimal temperature and buffer composition were adopted according to previous work.^30^ The choice of the above-mentioned cutinase relied on its excellent performance on other FDCA-based polymers and alternative 2,5-thiophenedicarboxylic acid-based polyesters, since 50 to 100% weight loss had been achieved for all tested films within 72 h of incubation.^31^

A first visual evaluation of the films hydrolyzed with Thc_Cut1 displayed different hydrolysis trends, clearly dependent on the PLA/PPeF ratio. Films with higher content of PLA (95–99% in PP5, PP3, and PP1) were more resistant to the enzymatic treatment, preserving their original aspect and transparency. Apart from being fragmented or not even detectable after the 6th day, the samples with 30 and 50 wt % PPeF content already showed a rougher and opaque surface on the first sampling after 72 h.

Incubation temperature and buffer proved to be functional, as optimized by Gamerith et al.^32^ Gravimetric analysis of pure PLA and PPeF samples recovered after hydrolysis showed a maximum of 20% of weight loss for PLA, while PPeF was completely degraded within the first three days of incubation (even though, given the same surface area, PPeF films were up to five times heavier due to their thickness).

**Figure 1:**
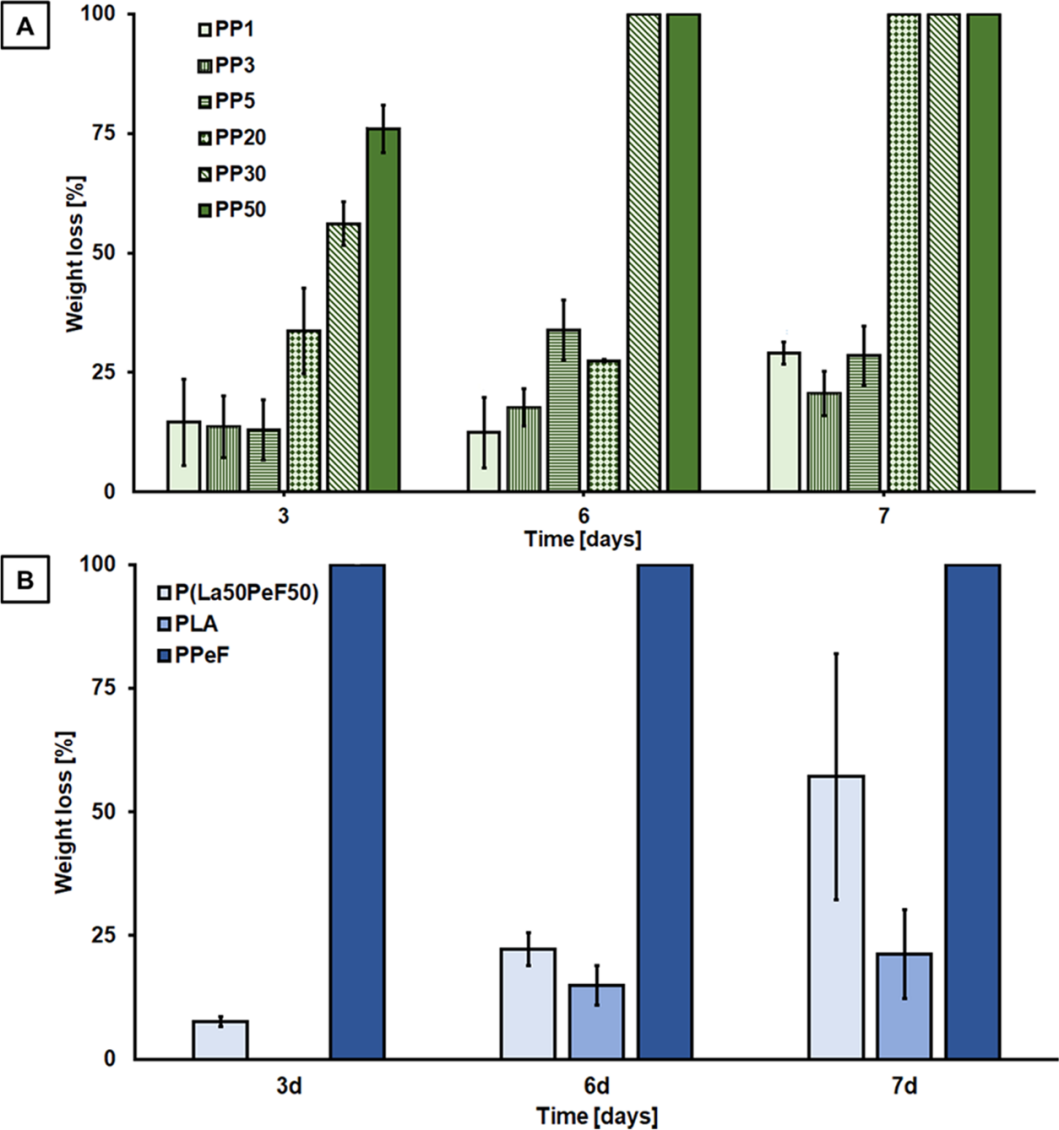
Weight loss of samples under investigation hydrolyzed with Thc_Cut1 after 3, 6, and 7 days of reaction time. (A) Physical blends with different PPeF content (ranging from 1 to 50 wt %). (B) Neat polymers and the P(LA50PeF50) block copolymer. The data shown are the average of triplicates. Physical blends indicated as PP1: PLA-PPeF 1; PP3: PLA-PPeF 3; PP5: PLA-PPeF 5; PP20: PLA-PPeF 20; PP30: PLA-PPeF 30; PP50: PLA-PPeF 50. Block copolymer P(La50PeF50).

Consistently, as reported in Figure 1A, the PLA blends with the highest PPeF content (PP30 and PP50) showed the highest weight loss of 56 and 76%, respectively, after 3 days of incubation. Besides being significantly higher than the value measured for PP20 (33% weight loss after 3 days), the obtained weight losses are also higher than the PPeF weight fraction, suggesting that a greater PPeF content also facilitated the hydrolysis of the PLA component. A reason for these circumstances was found in the enzyme specificity. PPeF was the preferred cutinase substrate, implying an increase of the whole polymer degradation consistently with a higher percentage of PPeF in the total blend composition. Likewise, increasing the PPeF proportion in the blend improved PLA hydrolysis since the polymeric matrix became plausibly less dense after the selective elimination of PPeF. Thus, PLA turned more accessible to enzyme attack. Complying with the former observations, enzyme specificity also justifies why the percentage of PPeF/PLA affected the hydrolysis trend. PP20 showed the quickest FDCA release after an initial lag, proving to be an optimal ratio between the substrate and applied concentration of the enzyme. For the P(LA50PeF50) block copolymer, a higher hydrolysis extent was measured (57% after 7 days) when compared to neat PLA (only 21% after 7 days). However, in contrast to the blends, the presence of furan moieties does not seem to enhance overall hydrolysis (Figure 1B). The higher resistance of P(LA50PeF50) to enzymatic hydrolysis is most likely related to the chemical covalent bond present between the rubbery and flexible PPeF blocks and the glassy and rigid PLA ones, these latter decreasing the susceptibility of PPeF moieties, and thus the copolymer, to enzymatic cleavage.

**Figure 2:**
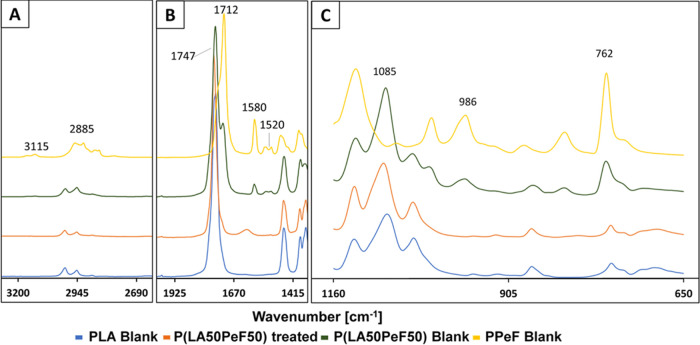
FT-IR ATR spectra of the P(LA50PeF50) block copolymer after enzymatic hydrolysis compared with the control. A: Zoom in on the 3200–2690 cm^–1^ region, B: zoom in on the 1925–1415 cm^–1^ region, and C: zoom in on the 1160–650 cm^–1^ region.

Surface analysis of the polymers was subsequently performed through both ATR-FT-IR and SEM. Given the sensitivity of the molecular bonds’ vibrations to infrared radiation, FT-IR can provide insight into the surface functional groups. The spectrum of the copolymer was of particular interest when compared to PPeF and PLA homopolymers. Figure 2 displays the three FT-IR spectra ranges in which the main changes occur.

The 3200–2690 cm^–1^ range corresponds to the region of the O–H stretching and =CH stretching mode of the furan ring. As shown in Figure 2A, both the not treated copolymer and PPeF present a weak-intensity doublet at 2855 cm^–1^ in addition to another almost superimposed signal. These signals correspond to the symmetrical CH stretching of C–H bonds (methylene of PPeF and the methyl group of lactic acid). After the hydrolysis, only the band at 3115 cm^–1^ and the second peak of the doublet at 2855 cm^–1^ were not detectable. These were uniquely associated with PPeF, while the remaining peaks are typical for PLA, as they could also be detected in the PLA blank curve.

The band at 1925–1415 cm^–1^ (Figure 2B) is associated with the range of the carbonyl bond stretching. In agreement with previous considerations on the O–H stretching, PPeF and the control copolymer showed some bands that are less appreciable in the treated sample and absent in the PLA film. These are mainly due to the aryl ester stretching of PPeF (1712 cm^–1^); as a consequence of the reduction of this band intensity, the 1747 cm^–1^ peak showed instead a proportional increase (since it corresponds to the aliphatic ester stretching). Additionally, an intensity reduction of the band at 1580 cm^–1^ was discernible upon enzymatic treatment. This is associated with the FDCA ring C=C stretching as well, which is not present in PLA and significantly reduced on the treated copolymer surface. The third range from 1160–650 cm^–1^ (Figure 2C) showed an accordant behavior since a notable decrease of =CH out-of-plane bending (of the disubstituted furan ring) could be detected (bands at 986 and 762 cm^–1^).

Overall, the greatest intensity reductions were recorded in PPeF-associated groups, particularly in the ester region, which is the main target of cutinase-catalyzed hydrolysis. Moreover, all of the highlighted data clearly substantiate the higher susceptibility of PPeF to enzymatic cleavage. Expectedly, after enzymatic hydrolysis, the spectrum of the copolymer became similar to that of PLA (eg. band at 1085 cm^–1^), whose main signals remained almost unaltered. Minor differences in fact were seen between the spectra of treated and control samples of PP1, PP3, and PP5 (see the SI, Figures S2–S7).

From SEM pictures, it was possible to elucidate the effective blend structure. In accordance with the results discussed above, the PPeF surface was the most affected by enzymatic treatment. For neat PLA and PP5, a homogeneous surface even at higher magnification (1000×) was seen. After 3 days of incubation, a uniform pattern of attack appeared on both surfaces (see the SI, Figures S8 and S9).

**Figure 3:**
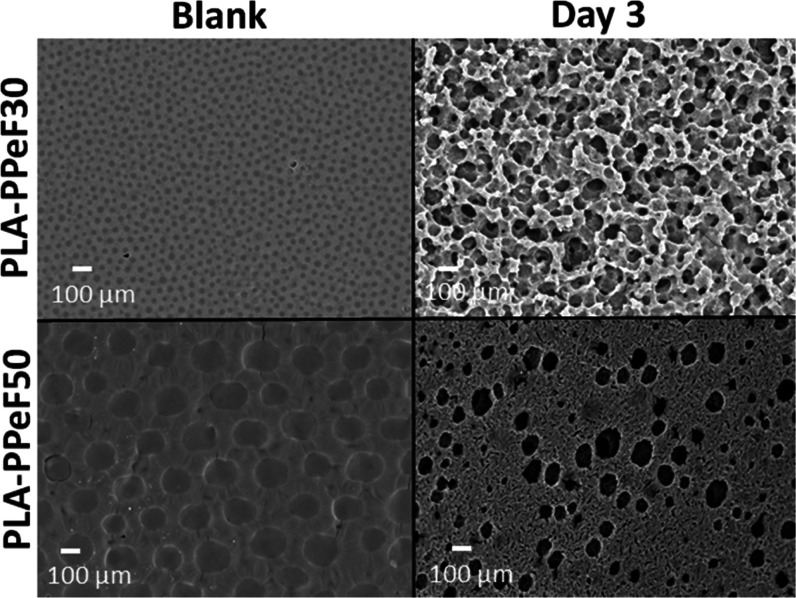
SEM imaging of PP30 (top) and PP50 (bottom) film blends enzymatically hydrolyzed for 3 days (right) and the respective controls (buffer only, left) at 1000× magnification.

It is interesting to notice how PPeF is actually embedded in the PLA matrix appearing as regular spherical patches with a diameter of ∼30 μm in PP30 and 140 μm in PP50. The higher the amount of PPeF, the larger the area of patches. After 3 days of treatment, the PPeF domains were almost gone, as visible in the micrographs at 1000× magnification. This is especially clear in PP30 and PP50 samples where, after the enzymatic treatment, deep holes and a rougher surface appear where the PPeF domains were located (Figure 3).

**Figure 4:**
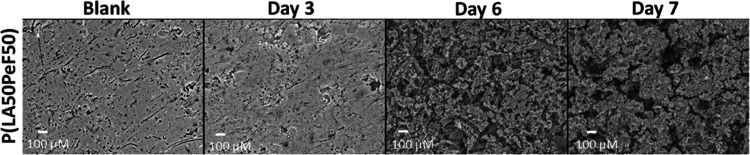
SEM imaging of P(LA50PeF50) enzymatically hydrolyzed for various time intervals and the respective controls (buffer only) at 1000× magnification.

The P(LA50PeF50) block copolymer showed instead an intermediate behavior (Figure 4). Even though the blank surface was not completely flat, likely due to the phase separation of the PLA and PPeF domains, changes induced by the enzyme treatment were visible. On the sixth or seventh day of incubation, an uneven distribution of degradation spots was seen. This would suggest a selective attack of the enzyme on the PPeF domains of the copolymer consisting of PPeF and PLA linked by diamine spacers.

### Characterization and Quantification of Released Products

The respective acid components (2,5-furandicarboxylic and lactic acid) released from the different polymers were quantified through HPLC analysis, exploiting different detection techniques. While the aromaticity of the furan ring was monitored through a UV detector, quantification of the linear lactic acid relied on a refraction index detector.

For the blends of PLA and PPeF, as well as for the P(LA50PeF50) copolymer, an increase in the concentration of the acids was measurable. Control reactions (incubation with buffer only) did not lead to any detectable release of acids, while the hydrolysis profiles (the relative ratio of released lactic acid and FDCA) of the treated samples were consistent with the known composition and observed weight loss reported above. The concentration of FDCA increased with the PPeF content in the blend: after 7 days, PP20, PP30, and PP50 gave, respectively, 0.14 mg, 0.18 mg, and 0.30 mg per 1 mg of the initial polymer. Consistently, the release of lactic acid was found to decrease with increased PPeF: for PP20, PP30, and PP50, 0.94 mg, 0.86 mg, and 0.53 mg per 1 mg of polymers were released, respectively. Moreover, the P(LA50PeF50) copolymer showed a lower amount of released products when compared to the blend with the same composition. Again, this is in agreement with the significantly lower weight loss seen for the copolymer when compared to the blend.

**Figure 5:**
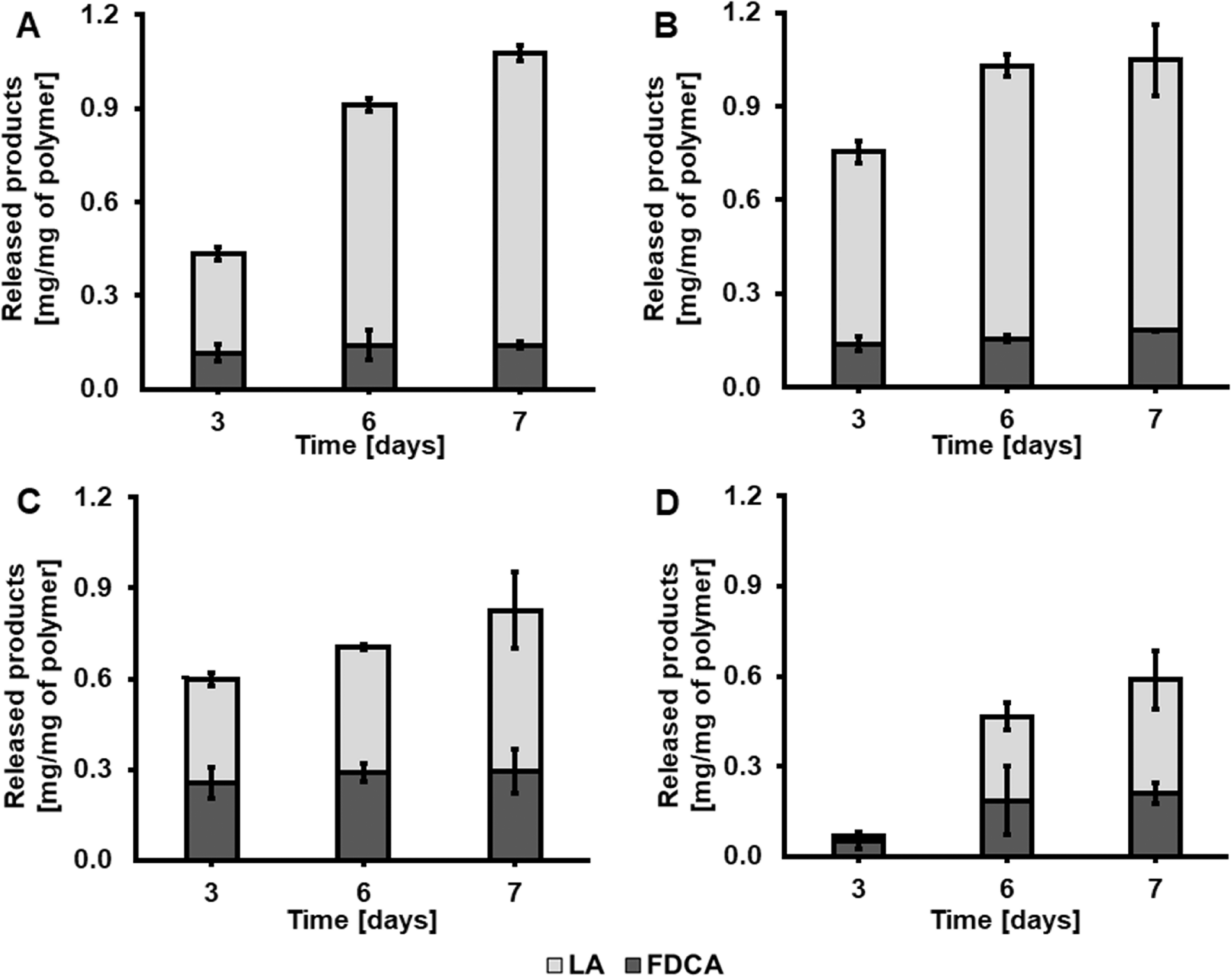
HPLC analysis of acids released from PLA/PPeF blends and the P(LA50PeF50) copolymer. Dark gray bars: 2,5-furandicarboxylic acid (FDCA); light gray bars: lactic acid (LA). (A) PP20; (B) PP30; (C) PP50; (D) P(LA50PeF50). The quantification of FDCA was the average of triplicate measurements and LA of duplicates.

Also based on the release of acids, the fastest hydrolysis rates were seen for PP20, PP30, and PP50 (as summarized in Figure 5).

Nuclear magnetic resonance was chosen to obtain insight into the structure of the polymers at a molecular level. The hydrolysis solution (buffer, enzyme, and remaining film) was lyophilized to remove all moisture and resolubilized in deuterated chloroform. Due to the peculiar environment surrounding each proton, their correspondent signals are shifted differently and can be plotted according to their chemical shift. This allows the assignment to a specific group and the integrated area can be used as an estimation of the relative signals’ ratio. The trend of this ratio can be used as a means to elucidate the PLA/PPeF ratio change upon enzymatic incubation.

**Figure 6:**
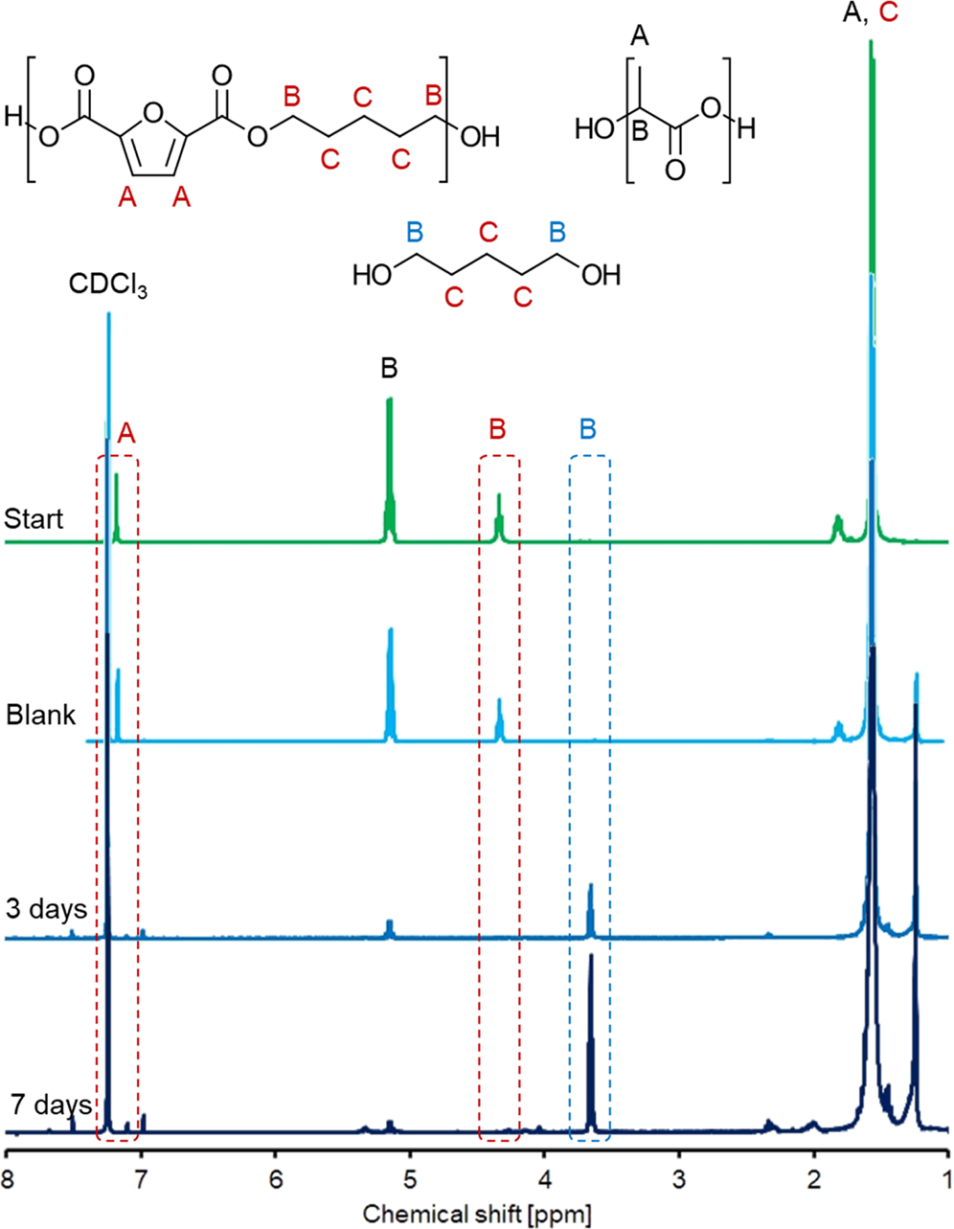
^1^H NMR spectra of PP30 at different stages of the enzymatic hydrolysis reaction when compared with the starting material (start, green) and the control reaction (blank, light blue). Integrated areas are reported in the SI, Tables S1 and S2.

Resonances detected at 7.2 ppm can be associated with furan ring protons (close to the CDCl_3_ signal at 7.26 ppm in Figure 6). The chemical shift of 1.8 ppm and the less intense signals at 4.3 ppm correspond, respectively, to the protons within the methylene chain and the external esterified CH_2_ protons of PPeF (C–O–CH_2_CH_2_CH_2_−). PLA instead shows signals at 1.56 and 5.15 ppm (CH_3_ and CH, respectively), and they can be therefore used as a reference for comparison.

In the hydrolyzed samples, new signals compared to the blank were detected (3.66 and 1.24 ppm) that are reasonably associated with the hydrolysis products. Specifically, CH_2_-OH protons would give resonance at 3.66 ppm and reveal an increase in free monomer (or PPeF end groups) that is consistent with successful depolymerization. At the same time, the relative ratio between PPeF and PLA correspondent groups confirms the greater susceptibility of PPeF to hydrolysis compared to that of PLA. It was, in fact, reduced from 0.3 to 0, indicating a complete degradation of the PPeF polymer into monomers as early as after 3 days. This effect was more evident for those samples with higher concentrations of PPeF, such as PP30 (Figure 6).

### Monomer Recovery

After enzymatic hydrolysis and removal of the residual films, the remaining solution contained enzymes, the buffer, and the oligomers/monomers released from the polymer films. In particular, the recovery of FDCA for possible recycling concepts was investigated. In order to convert FDCA into dipotassium salt, a pH shift crystallization was exploited.^33^ Indeed, lowering pH 8 (incubation buffer) to pH 1.2 (significantly lower than the pKa of both −COOH groups of the molecule) by means of concentrated HCl addition led to the precipitation of a white powder. From a first evaluation of the obtained precipitate, it was deduced that some part was still in its salt form (see the SI, Figure S10). Therefore, a further acidification step of the separated pellet at pH 1 using a higher temperature (80 °C) was used to separate the diacid form (according to the suggested protocol^34^). The final recovery of the diacid was confirmed via TGA (see the SI, Figure S11). The temperature at which the mass loss of recovered FDCA reached 10% (10% decomposition temperature or *T*_d_10) and 50% (50% decomposition temperature 50 or *T*_d_50) of the initial weight is almost superimposable with the thermal behavior of pure FDCA (*T*_d_10: 285 vs 300 °C for pure and recovered FDCA, respectively; *T*_d_50: 340 °C for both).

The separation of FDCA from the liquid fraction was additionally proven through HPLC quantification. The hydrolysis of 1.5 g of the PPeF homopolymer in 100 mL of 5 μM Thc_Cut1 solution led to a measured FDCA concentration of 88 mM. This value decreased to 0.43 mM after the first acid precipitation and crystallization, while after the second resuspension and acidification, there was no detectable soluble FDCA (residual solution HPLC spectrum in SI Figures S12 and S13). At the final stage of hydrolysis, the homopolymer hydrolysate is completely depolymerized into monomers, as suggested by HPLC-quantified FDCA, that remain soluble in solution at the incubation buffer conditions. The separated FDCA appeared white and clean from impurities, appearing very similar to the commercially available acid (see the SI, Figure S14). Given the ratio between the two monomers’ (FDCA and EG) molecular weights, a theoretical maximum amount of recoverable FDCA can be estimated. 1.07 g of FDCA would result upon complete depolymerization of 1.5 g of PPeF. The final processed powder accounted for 0.75 g by manual weighing, therefore ∼75% of the expected maximum theoretical yield.

### Resynthesized PPeF (R-PPeF) Characterization

The synthesis was carried out as shown in Figure S15. The polymer yield was >90%. Molecular characterization through FT-IR was carried out; the comparison between PPeF (neat polymer) and R-PPeF spectra confirms the good control of the synthesis process as the two spectra practically overlapped (see Figure S16, SI). ^1^H NMR analysis corroborates the chemical structure of R-PPeF, evidencing the presence of the furan protons at 7.19 ppm, the −OCH_2_– at 4.35 ppm, and methylene groups in the region between 1.9 and 1.5 ppm. The signal of the −CH_2_–OH proton is also detectable at 3.70 ppm. Two additional signals having a negligible intensity (2–3%) at 4.10 and 3.42 ppm were detected. It is worth highlighting that the functional properties of the resynthesized polymer are not compromised by this slight variation with respect to the neat PPeF polymer (Figures S17 and S18 in the SI).

GPC brings evidence of the possibility of resynthesizing PPeF with a high M_n_ value and good polydispersity index. As summarized in Table 1, resynthesized PPeF shows analogous properties when compared to the original PPeF. DSC confirms a similar amorphous nature, and also thermal stability is maintained high.

**Table 1:** Summary of GPC, DSC, TGA, and Tensile Tests Performed on Resynthesized PPeF in Comparison with the Neat PPeF Material

	GPC		DSC		TGA		tensile test		
	*M*_n_^a^	PDI^b^	*T*_g_ (°C)	Δ*C*_p_ (J/°C g)	*T*_max_ (°C)	*T*_onset_ (°C)	*E* (MPa)^c^	σ_B_ (MPa)^d^	ε_B_ (%)^e^
PPeF	29,600^25^	2.4^25^	13^25^	0.394^25^	414^25^	392^25^	9 ± 1	6.1 ± 0.5^25^	1050 ± 200^25^
R-PPeF	24,600	2.3	10	0.495	396	375	6 ± 2	2.0 ± 0.5	1400 ± 200

a Number-average molecular weight.

b Polydispersity index.

c Elastic modulus.

d Stress at break.

e Strain at break.

Furthermore, as concerns the mechanical response, R-PPeF displays a very similar behavior with respect to neat PPeF. The elastic modulus, elongation, and stress at break do not present appreciable variations.

## Conclusions

Despite many researchers focusing on alternative plastics in the last decade, still, 99% of produced plastics in use are not bio-based or biodegradable. On the way to the establishment of a circular plastic industry, it is necessary to prove competitive production processes and concomitantly biodegradability/recyclability properties. Especially in food packaging applications, requirements are stringent. Food waste is another great concern of our era; therefore, it is necessary to provide packaging solutions, which, in addition to being sustainable, must ensure food preservation and protection. Moreover, food packaging is the least reusable and one of the most short-term plastics since the consumer would immediately discard it, and thus the possibility of integrating it into the recycling chain is an urgent need.

The combination, by blending or copolymerization, of different bio-based polymers such as PLA and PPeF represents an attractive strategy to produce sustainable and performant packaging, compensating the shortcomings of each single polymer.

Therefore, this work deals with a first evaluation of the susceptibility of new PLA/PPeF blends and block copolymers to enzymatic hydrolysis. On the one hand, hydrolysis by extracellular enzymes is the first step of biodegradation; on the other hand, enzymes have high potential in modern recycling strategies. Quantification of weight loss and released hydrolysis products demonstrated a faster depolymerization of PLA/PPeF blends compared to block copolymers, and the higher the rate of the process, the higher the PPeF content. In particular, those with a PPeF amount higher than 20 wt % were completely hydrolyzed. Interestingly, the P(LA50PeF50) block copolymer was less susceptible to enzymatic hydrolysis than the blend with the same composition. This confirms the higher susceptibility of FDCA-based polymers to esterase activity when compared to PLA. The preferential attack could be at least partially explained by the polymer structure, in particular with the PPeF segment that guarantees higher flexibility of the chain and easier enzyme accessibility to the polymer’s domains.

Besides the effective selective enzymatic depolymerization of furan-containing moieties, the method proposed also allows the recovery of pure FDCA, the more expensive monomer. Recovered FDCA has been repolymerized in a very efficient way, obtaining PPeF with the same solid-state properties as the neat homopolymer.

Selective hydrolysis places another important plus point on these novel formulations. Developing a handle eco-friendly method to recover highly pure monomers from recycled materials constitutes an impressive perspective for an already promising new generation of polymers.

## Supporting Information Available

Calibration curves, FT-IR spectra, SEM images, ^1^H NMR spectra, TGA analysis, HPLC chromatograms, and strain–stress curves (PDF)
