# Supplementary material for: On the Selective Enzymatic Recycling of Poly(pentamethylene 2,5-furanoate)/Poly(lactic acid) Blends and Multiblock Copolymers
Source: ACS Sustain Chem Eng. 2023 Jun 16;11(26):9751–60. doi: 10.1021/acssuschemeng.3c01796 (PMC10324456; doi:10.1021/acssuschemeng.3c01796)
Supplement: Supplementary file 1 — sc3c01796_si_001.pdf [file sc3c01796_si_001.pdf]

Electronic Supplementary Information

On the selective enzymatic recycling of poly(pentamethylene 2,5-furanoate)/poly(lactic acid) blends and multiblock copolymer

Chiara Siracusa[1], Felice Quartinello[1,2], Michelina Soccio[3,4,*], Mattia Manfroni[3], Nadia Lotti[3,4,5], Andrea Dorigato[6], Georg M. Guebitz[1,2], Alessandro Pellis[1,2,7,*]

[1] *acib GmbH, Konrad-Lorenz-Strasse 20, 3430 Tulln an der Donau, Austria.*

[2] *Institute of Environmental Biotechnology, University of Natural Resources and Life Sciences Vienna Konrad-Lorenz-Strasse 20, 3430 Tulln an der Donau, Austria.*

[3] *Department of Civil, Chemical, Environmental and Materials Engineering (DICAM), University of Bologna, Italy*

[4] *Interdepartmental Center for Industrial Research on Advanced Applications in Mechanical Engineering and Materials Technology, CIRI-MAM, University of Bologna, Bologna, Italy.*

[5] *Interdepartmental Center for Agro-Food Research, CIRI-AGRO, University of Bologna, Bologna, Italy.*

[6] *Department of Industrial Engineering and INSTM Research Unit, University of Trento, Trento, Italy.*

[7] *Department of Chemistry and Industrial Chemistry, Università degli Studi di Genova, Via Dodecaneso 31, 16146 Genova, Italy*

* Correspondence to: Dr. Alessandro Pellis, email: alessandro.pellis@unige.it and Dr. Michelina Soccio: m.soccio@unibo.it

Number of pages: 14

Number of schemes: 1

Number of figures: 19

Number of tables: 3

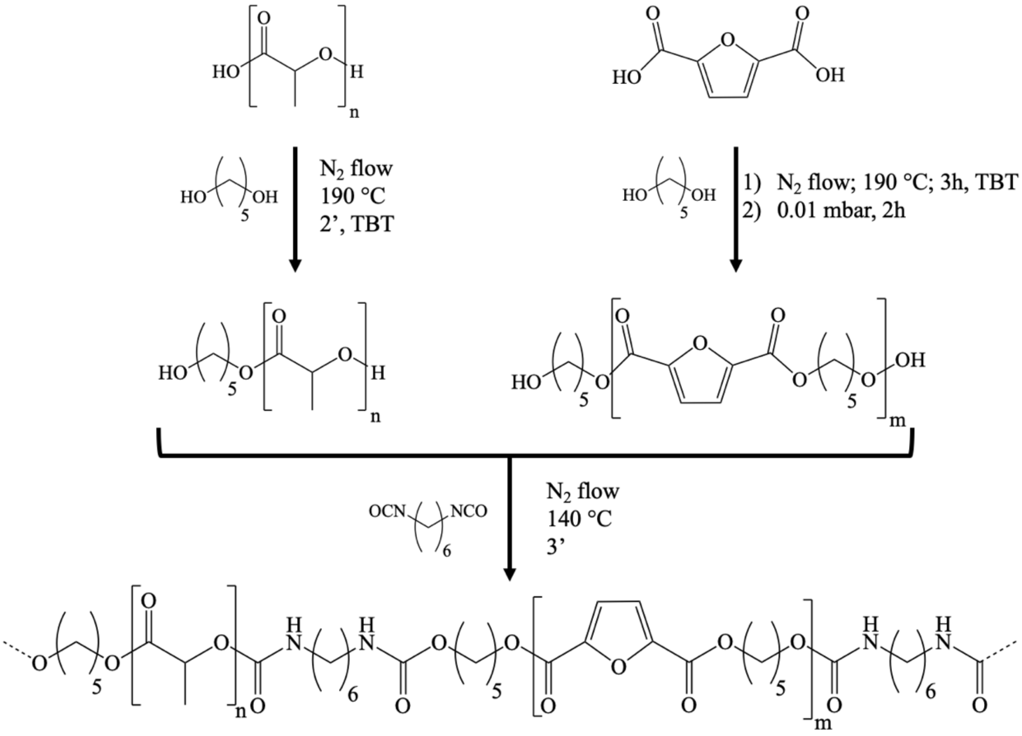

Scheme S1: Schematic representation of block copolymer synthesis.

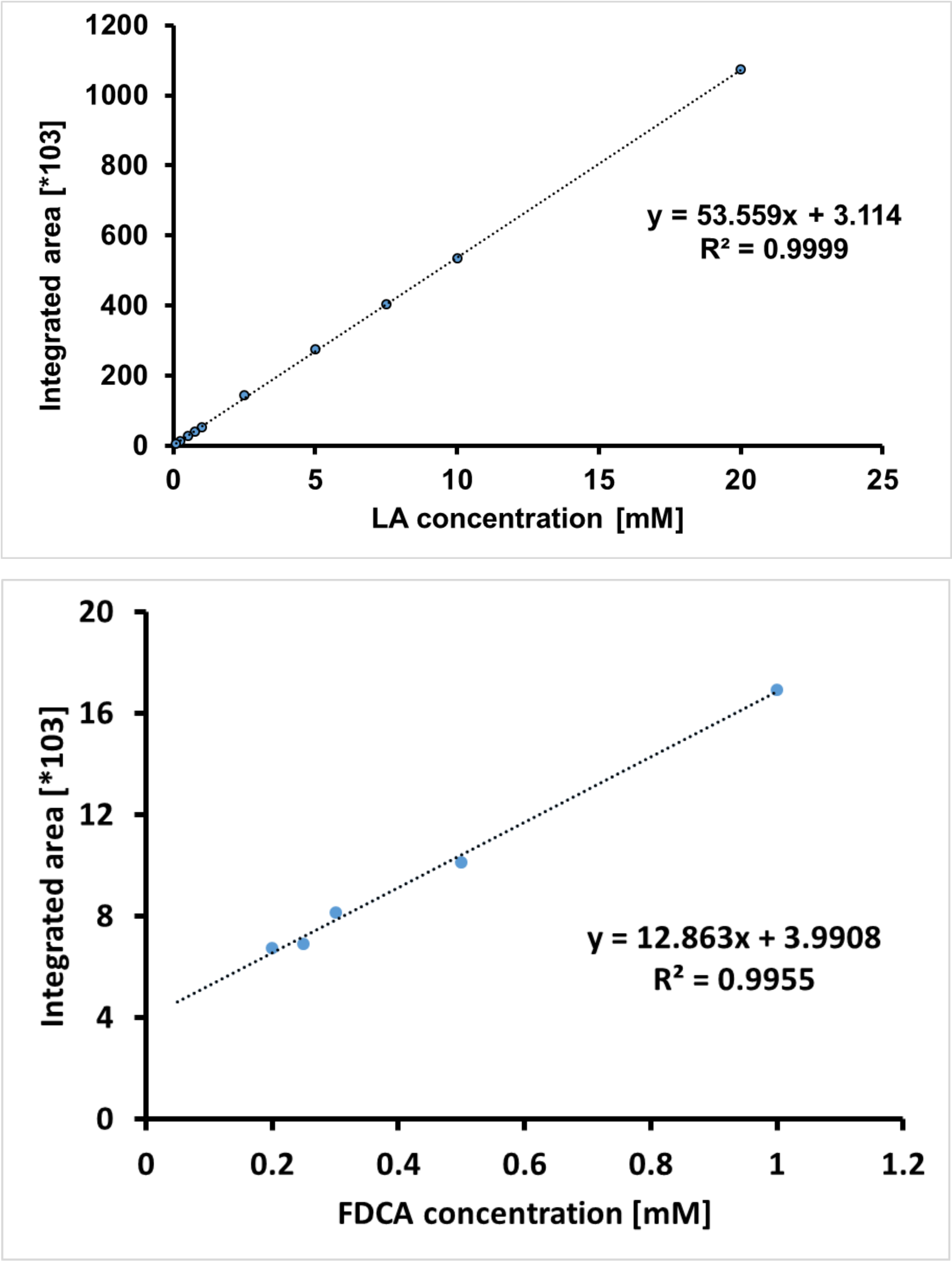

Figure S1. A) Calibration curve for LA concentration determination. B) Calibration curve for FDCA concentration determination.

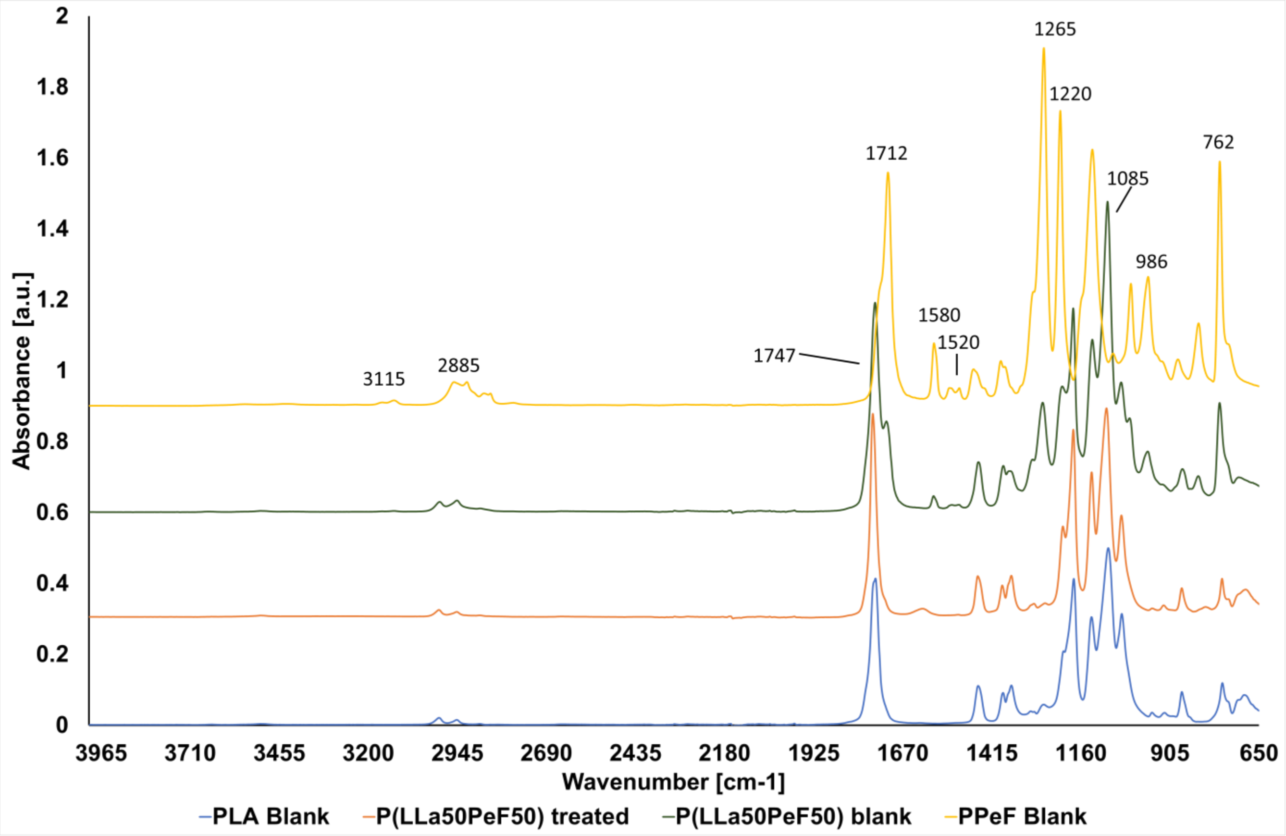

Figure S2. Full FT-IR spectrum of compared PLA and PPeF homopolymers with (P(LA50PeF50)).

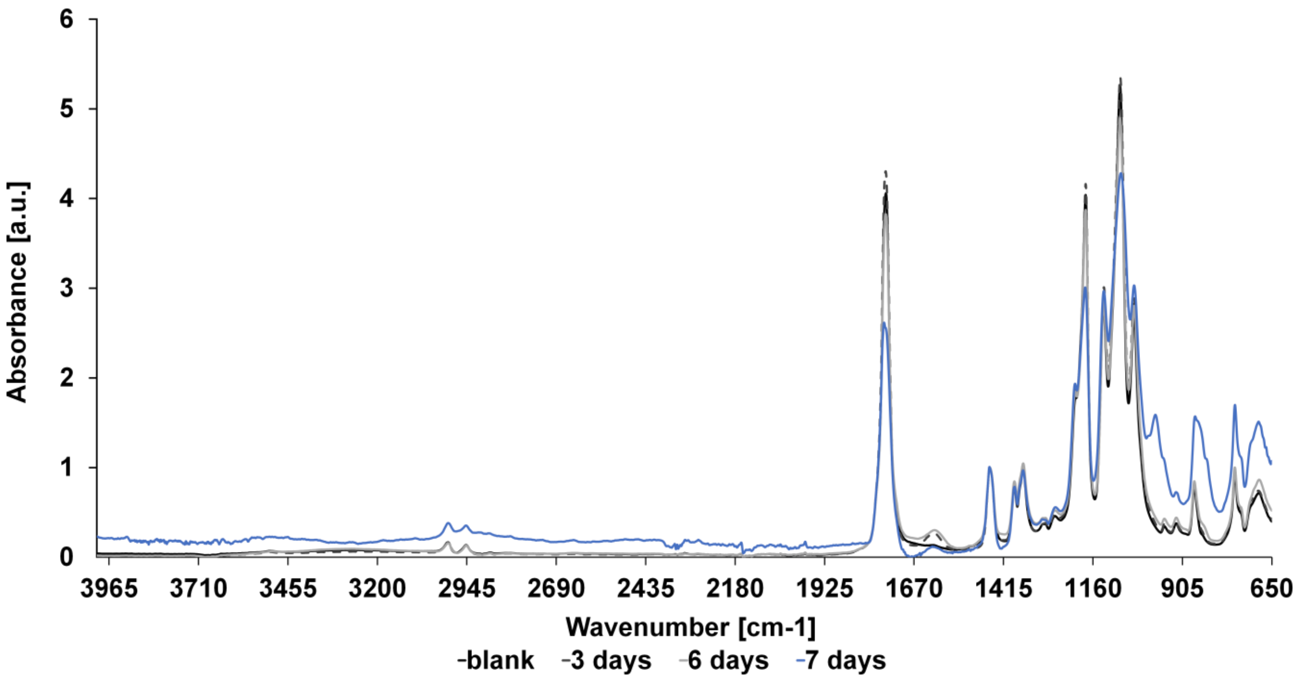

Figure S3. Full FT-IR spectra of PLA at different time points.

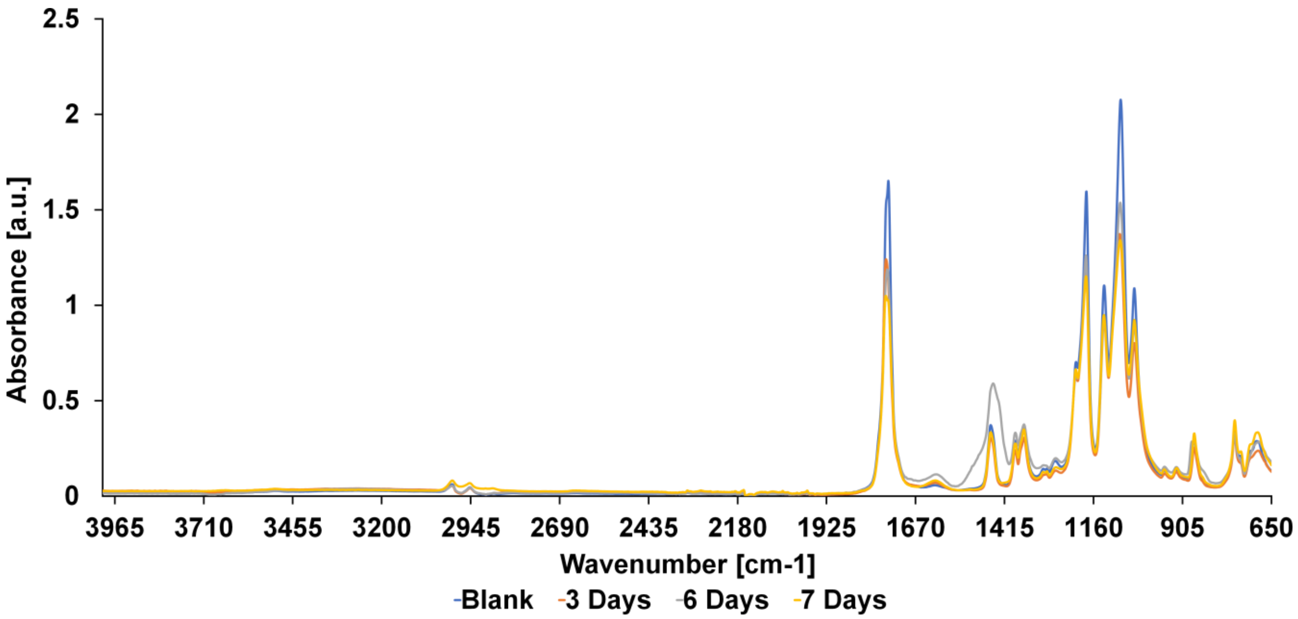

Figure S4. Full FT-IR spectrum of PP1 at different time points.

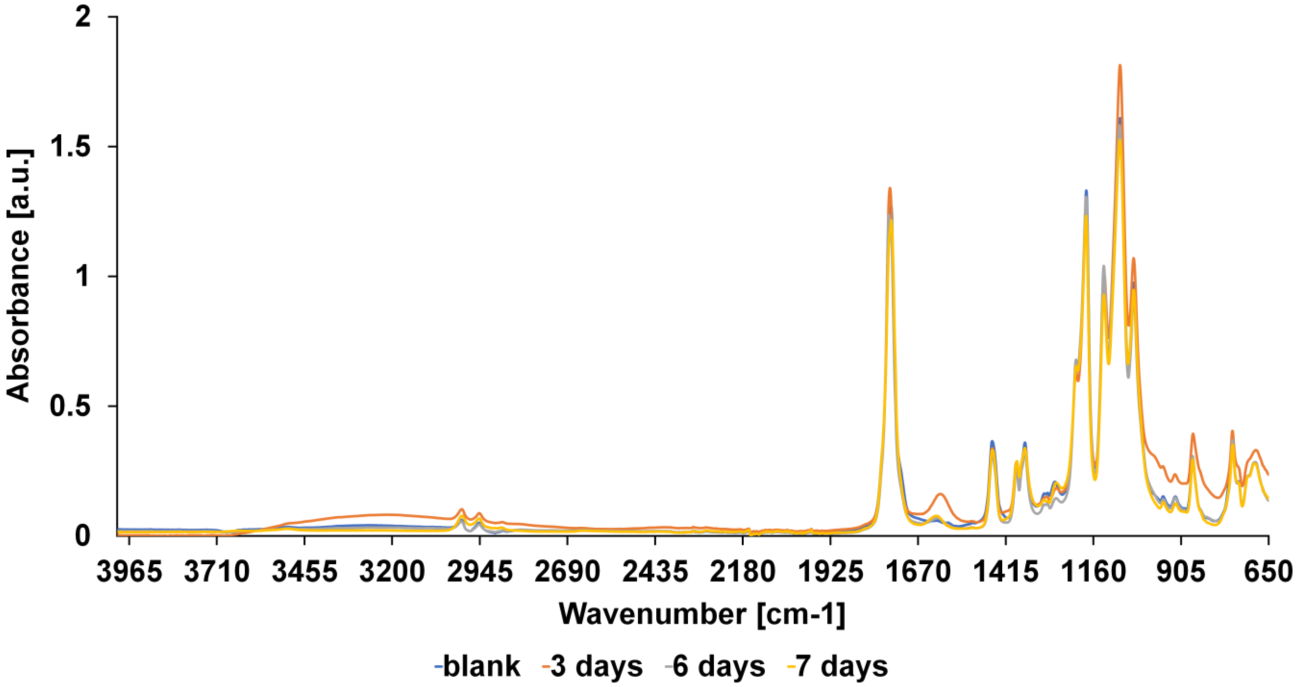

Figure S5. Full FT-IR spectrum of PP3 at different time points**.**

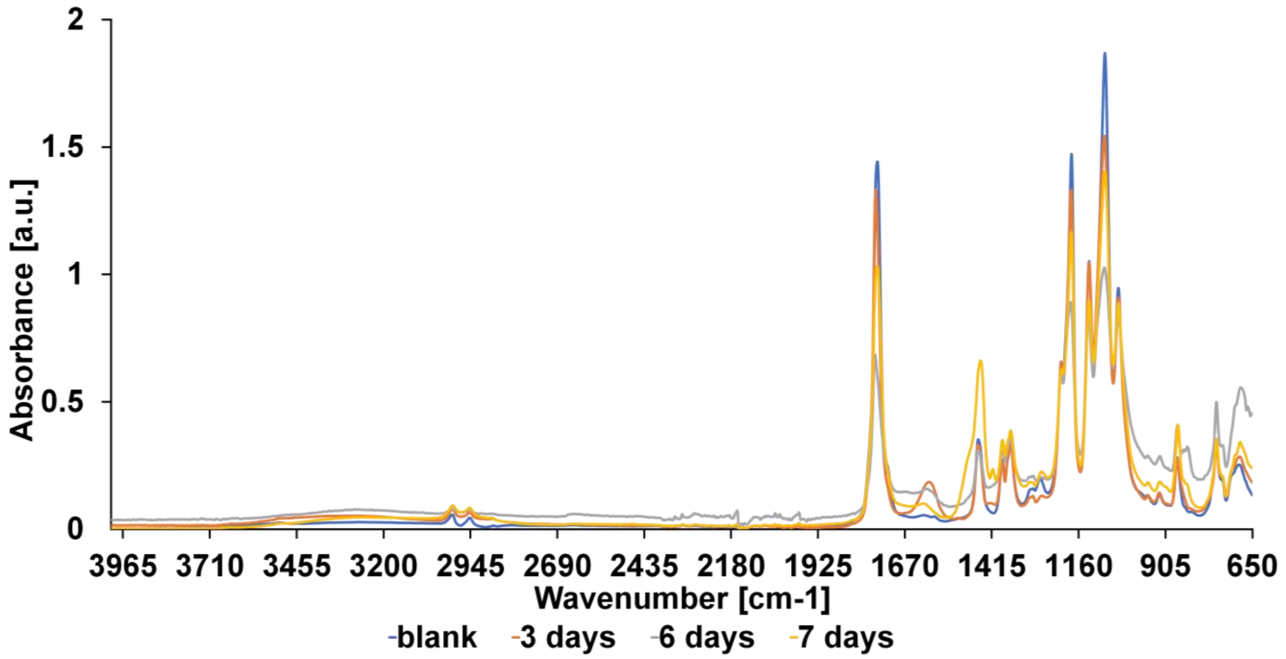

Figure S6. Full FT-IR spectrum of PP5 at different time points.

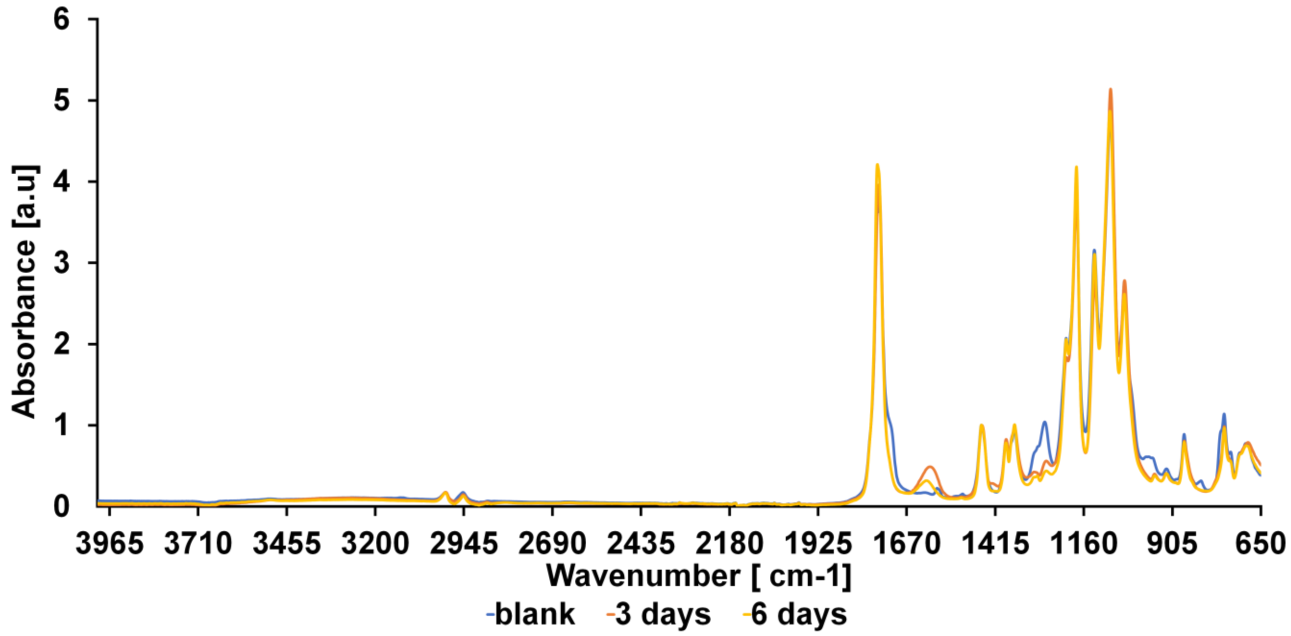

Figure S7. Full FT-IR spectrum of PP20 at different time points**.**

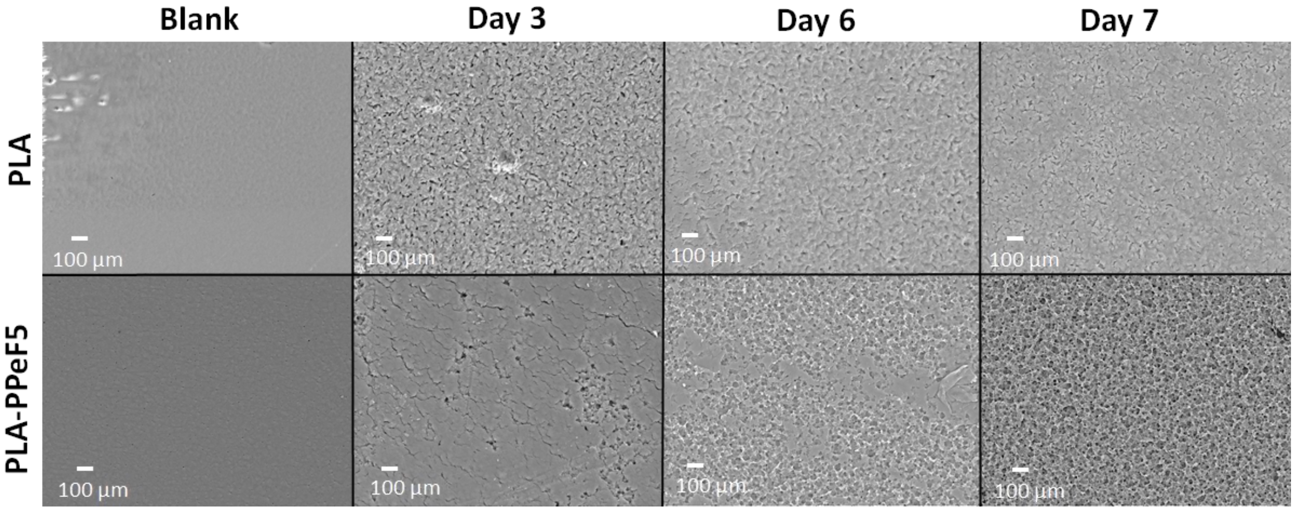

Figure S8. SEM imaging of PLA and PLA-PPeF5 controls (blank) and different reaction time points, at a 1000X magnification.

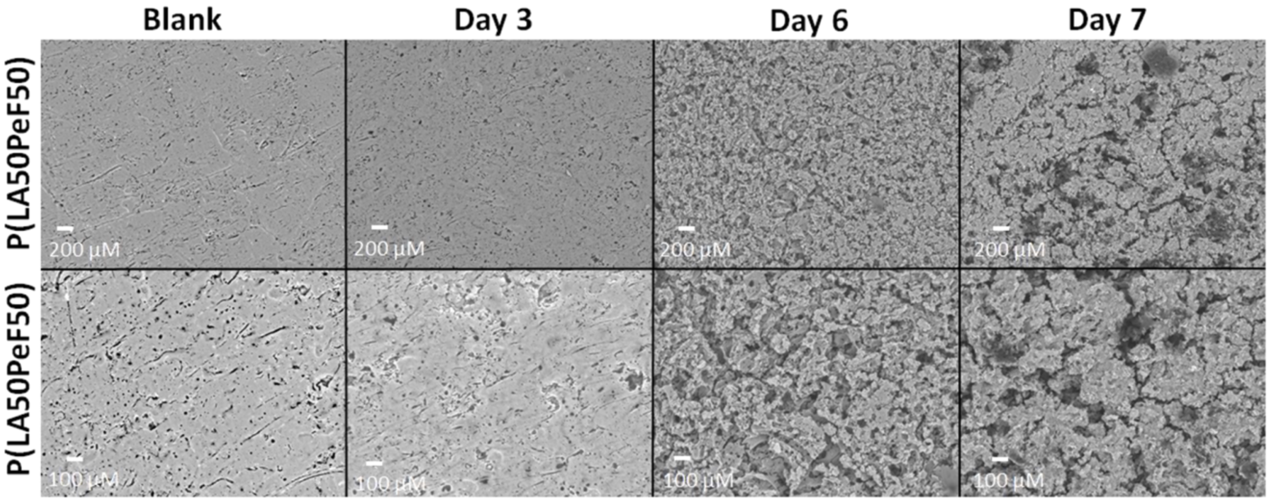

Figure S9. SEM imaging of (P(LA50PeF50)) controls and different reaction time points at 500 and 1000X magnifications.

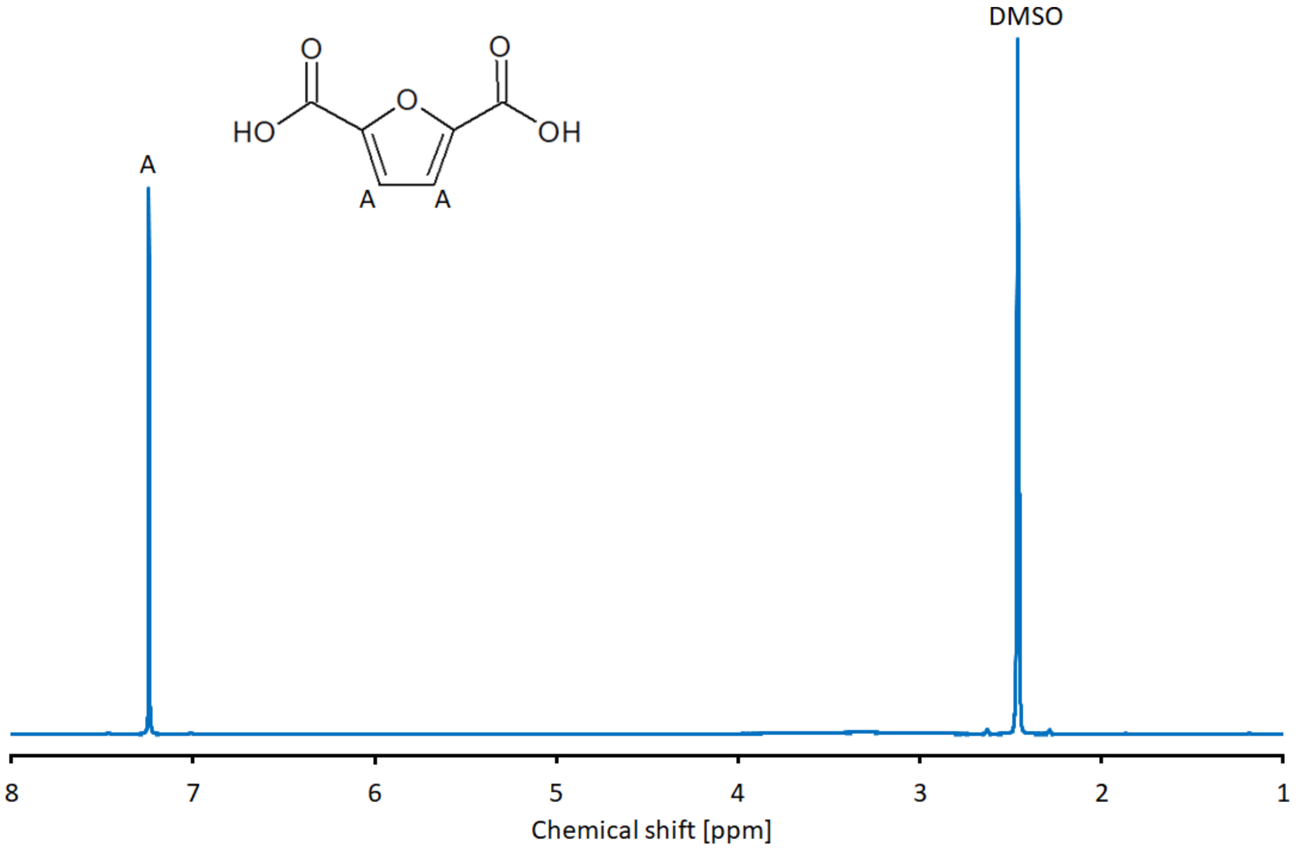

Figure S10. ^{1}H-NMR spectrum of the recovered FDCA. DMSO-d6 was used as NMR solvent.

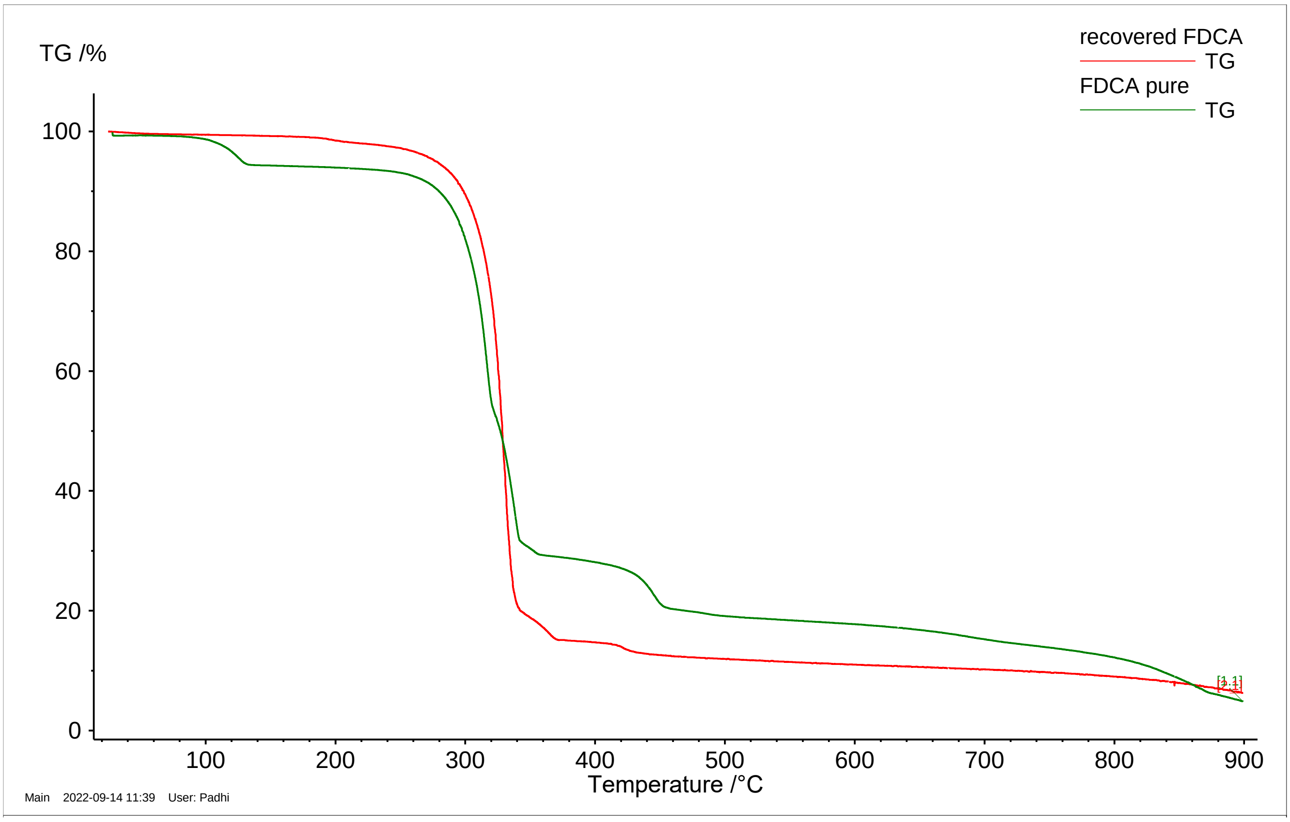

Figure S11. Thermogravimetric analysis of recovered FDCA (red line) compared to pure commercial FDCA (green line).

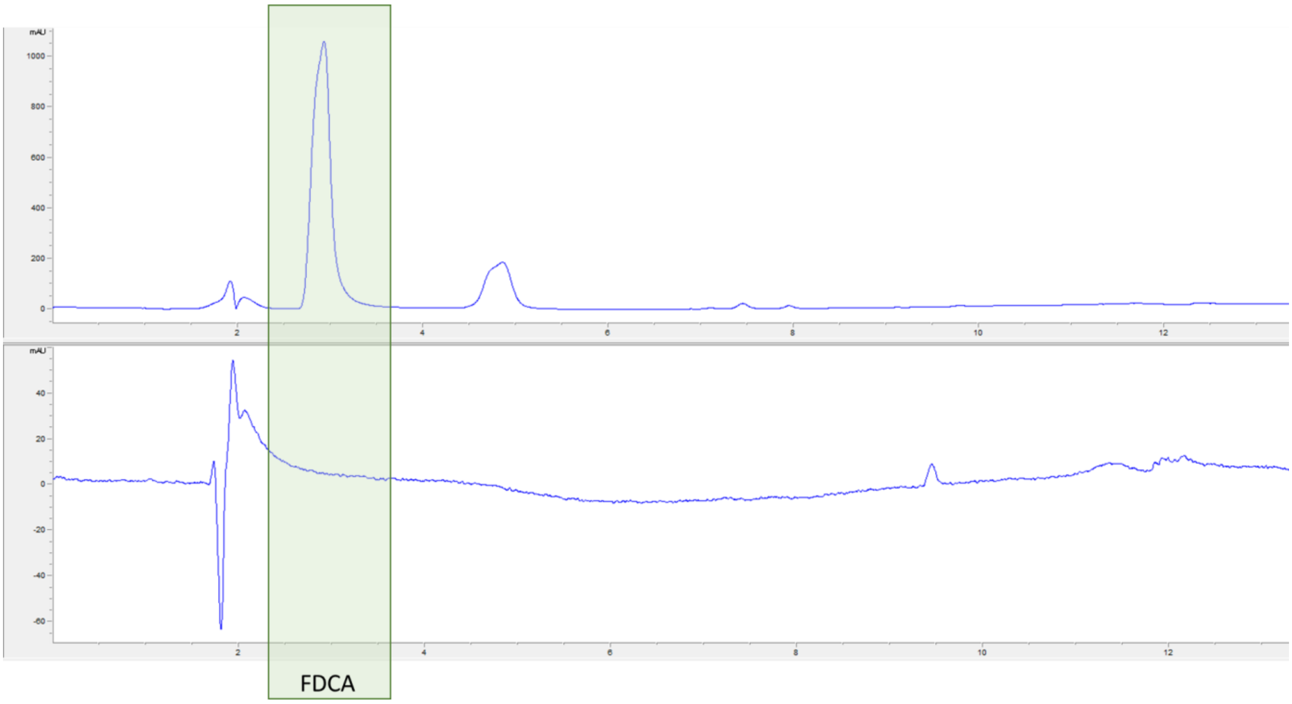

Figure S12. UV-HPLC chromatogram of PPeF hydrolysate before and after the precipitation via acidification. Retention time of FDCA: 2.7 min.

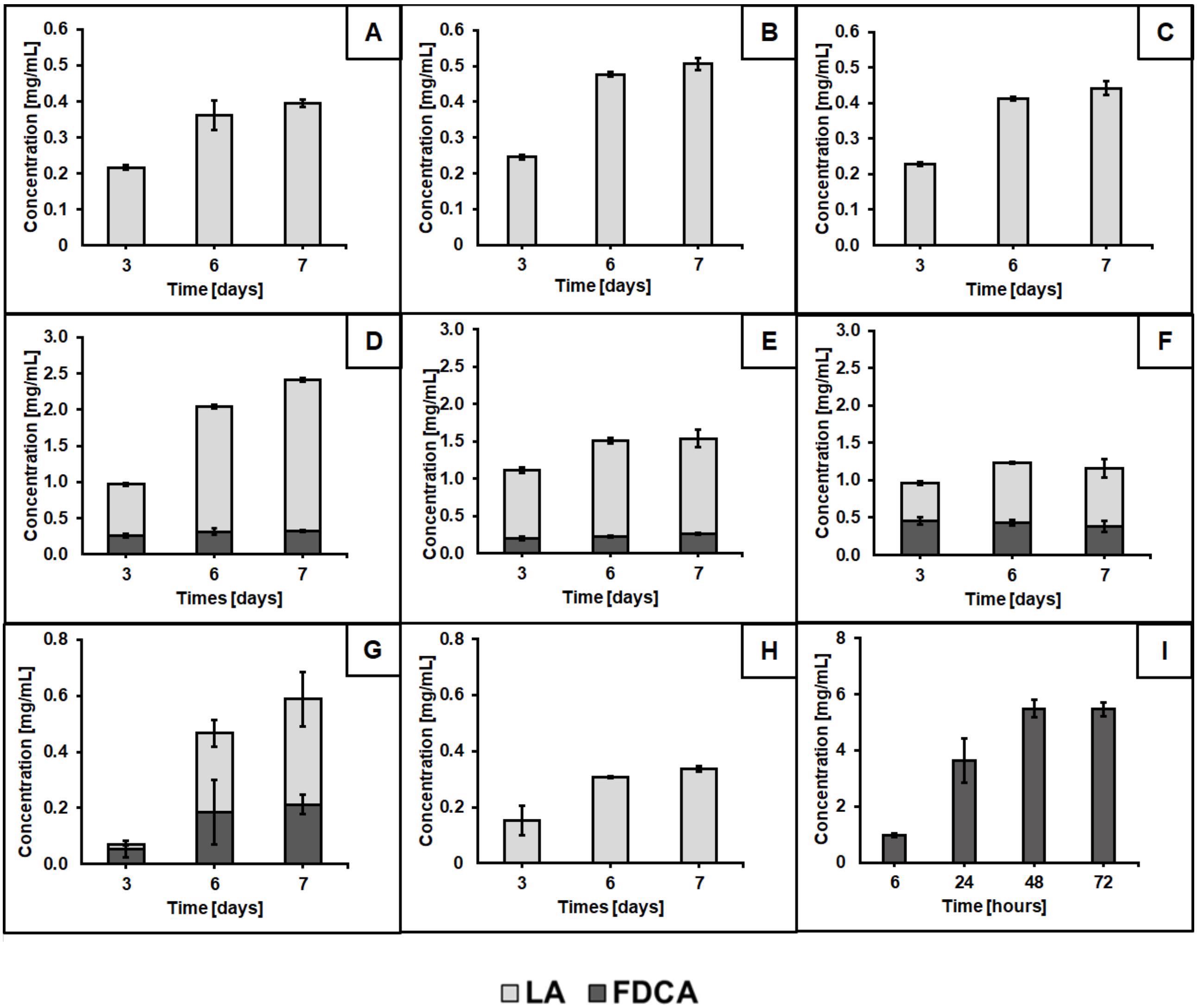

Figure S13. Concentration (mg/mL) of released monomers (FDCA and LA) in A. PP1, B. PP3, C. PP5, D. PP20, E. PP30, F. PP50, G. (P(LA50PeF50)), H. PLA, I. PPeF.

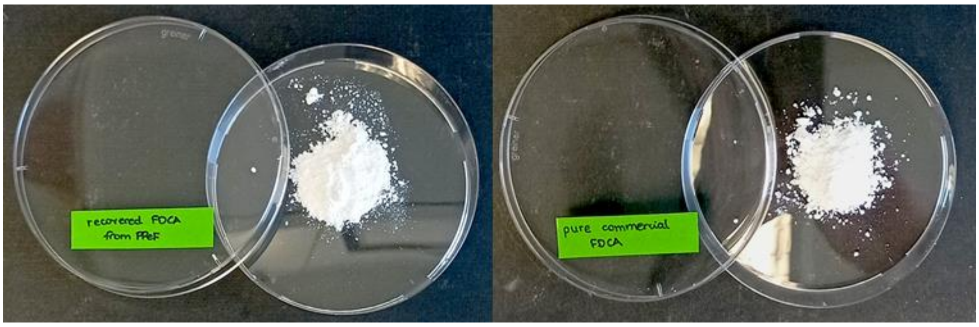

Figure S14. Comparison between the FDCA recovered from PPeF (left) and pure commercial FDCA (right).

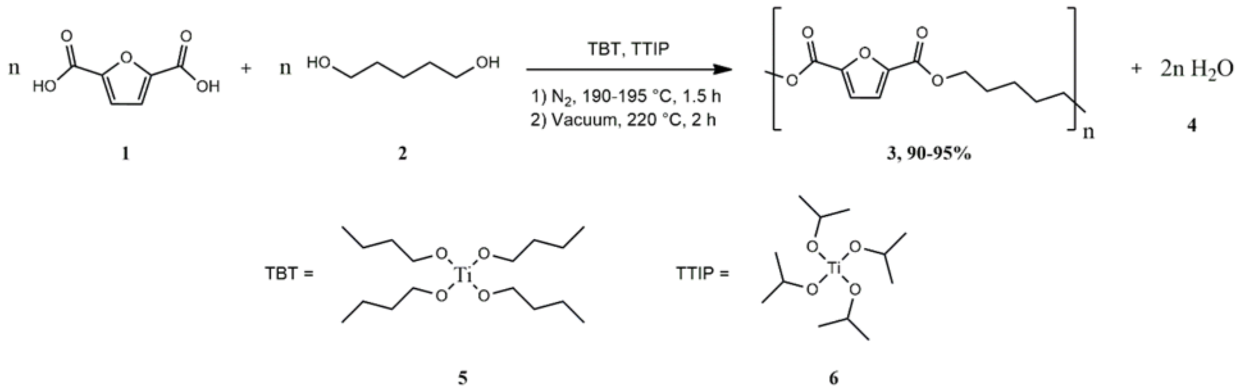

Figure S15. Reaction scheme for resynthesis of PPeF (R-PPeF). R-FDCA (Recovered-FDCA) (1), 1,5-Pentanediol (PD) (2), R-PPeF (3), titanium tetrabutoxide (TBT) (5), titanium isopropoxide (TTIP) (6)

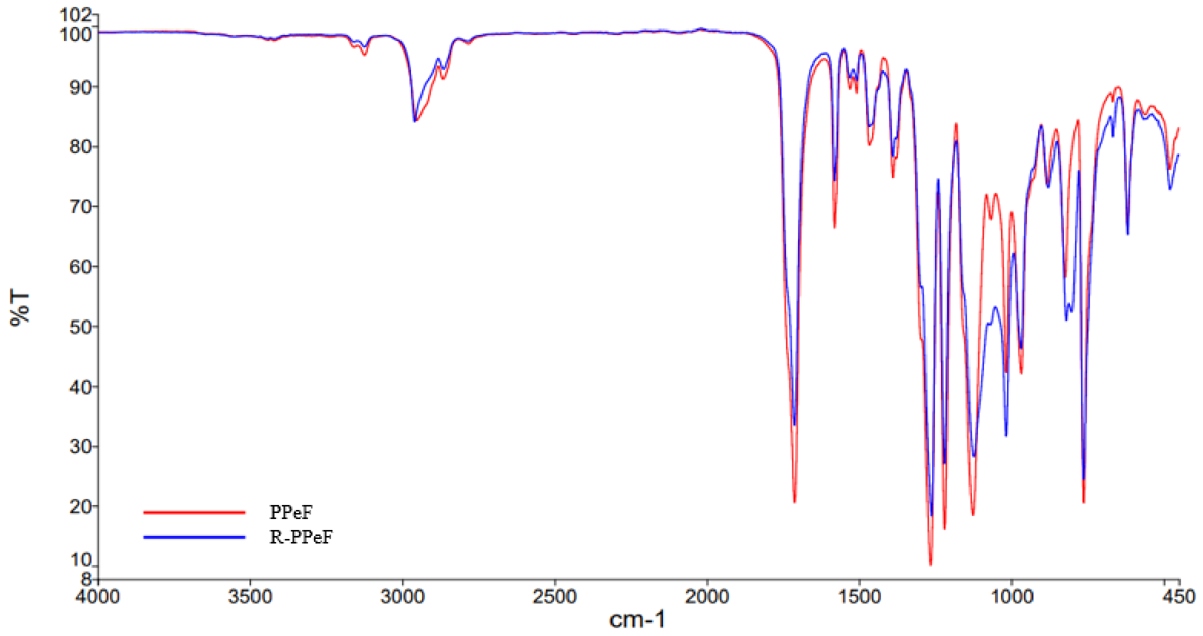

Figure S16. Comparison between FTIR spectra of PPeF (red) and R-PPeF (blue).

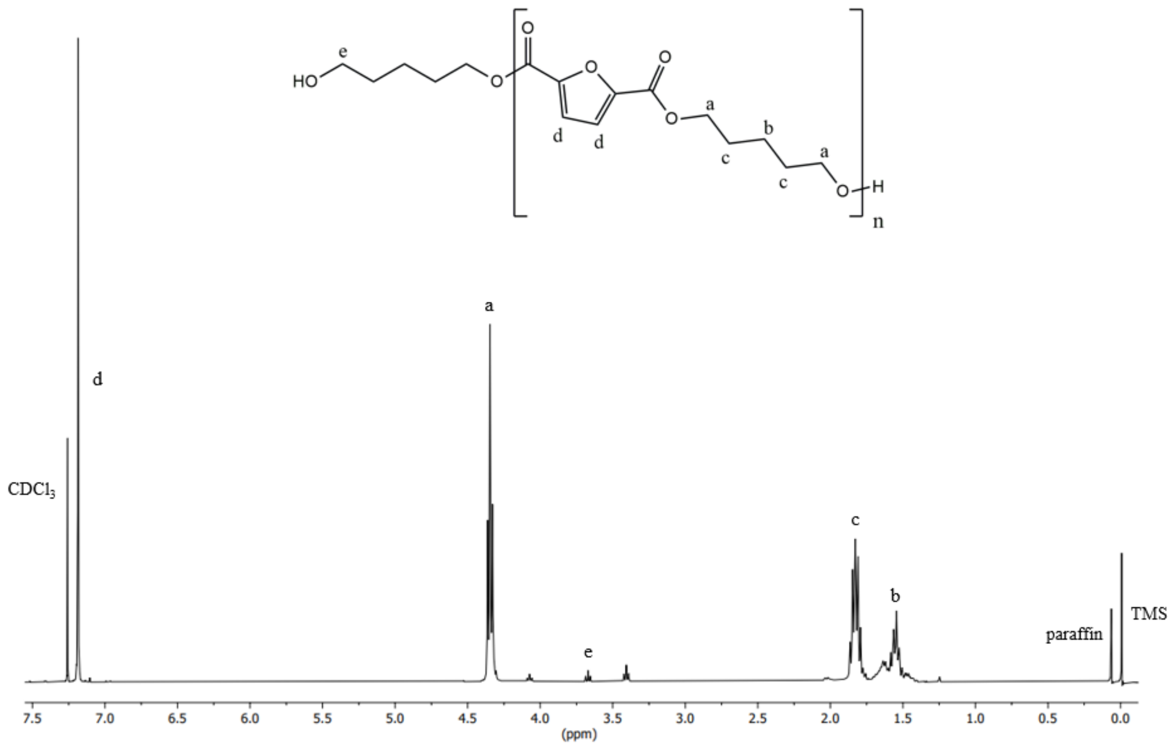

Figure S17. ^{1}H-NMR (400 MHz, $CDCl_3$) of R-PPeF.

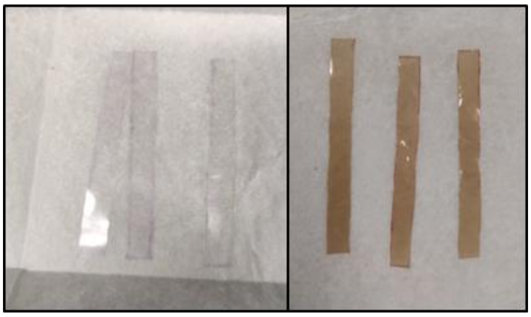

Figure S18. Samples of neat PPeF (left) and R-PPeF (right) used for stress/strain test (5.0x0.5 cm).

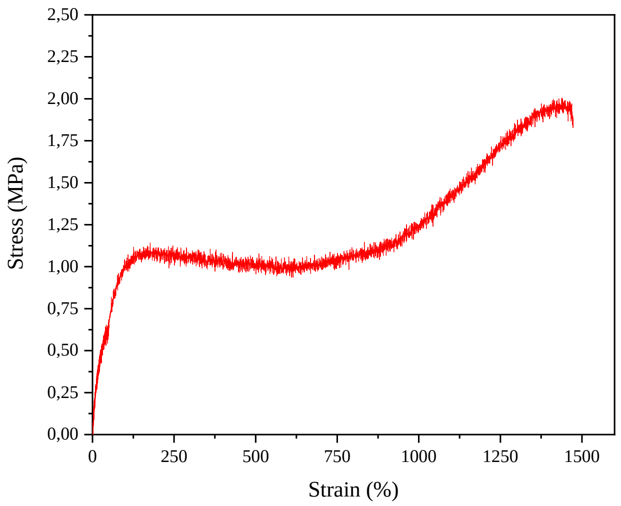

Figure S19. Representative stress/strain curve of R-PPeF.

Table S1. ^{1}H-NMR integrated area at the different PP30 hydrolysis time points.

			Integrated area		
**ppm**	**Assignment**	**Nr of protons**	**PLA-PPeF 30 Blank**	**PP30 3 days**	**PP30 7 days**
7.20	Aromatic protons FCDA	2	0.20	0.19	-
5.15	CH PLA	1	1	1	1
4.30	CH_2 PPeF	2	0.43	-	0.02
3.66	CH_2 PPeF monomer	2	-	2.57	0.02
1.80-1.56	CH_3 PLA+CH_2 PPeF	3+4	5.14	6.31	5.35

Table S2. ^{1}H-NMR integrated area in the different PP5 hydrolysis time points.

			Integrated area		
**ppm**	**Assignment**	**Nr of protons**	**PLA-PPeF 5 Blank**	**PP5 3 days**	**PP5 7 days**
7.20	Aromatic protons FCDA	2	0.02	0.01	0.01
5.15	CH PLA	1	1	1	1
4.30	CH_2-$\underline{CH_2}$-OC=O PPeF	2	0.04	0.03	0.02
3.66	CH_2 PPeF monomer	2	0.01	0.02	0.02
1.80-1.56	CH_3 PLA+CH_2 PPeF	3+4	4.04	6.96	5.00

Signals integrated areas are related to the CH of PLA, since it´s present in all samples. Some signals appear in the treated samples, being not detectable in the blanks: namely 1.24 ppm related peak, that could be associated to a hydrolysis product.

4.3 ppm and 5.15 ppm were chosen as reference signal respectively for PPeF and PLA, since they could be uniquely associated to those molecules.

Ratio of blanks correspond to the expected one, based on known starting composition.

The ratio PPeF/PLA (calculated on the signals above mentioned) decreases progressively along the proceeding incubation. The reduction rate is consistent with the ratio of PPeF present in the blend: PP30, as shown in table S1, results to be zero after the treatment.

Table S3. Data of synthesis of R-PPeF

	**M_W (g/mol)**	**n (mol)**	**Weight (g)**
**1**	156.09	0.0031	0.484
**2**	104.15	0.0124	1.291
**5**	340.32	/	200 ppm
**6**	284.22	/	200 ppm
